# Transportome-wide engineering of *Saccharomyces cerevisiae*

**DOI:** 10.1016/j.ymben.2021.01.007

**Published:** 2021-03

**Authors:** Guokun Wang, Iben Møller-Hansen, Mahsa Babaei, Vasil D'Ambrosio, Hanne Bjerre Christensen, Behrooz Darbani, Michael Krogh Jensen, Irina Borodina

**Affiliations:** The Novo Nordisk Foundation Center for Biosustainability, Technical University of Denmark, 2800, Kongens Lyngby, Denmark

**Keywords:** Transporter protein, Cell factory, Metabolic engineering, Betaxanthins, Muconic acid, Protocatechuic acid

## Abstract

Synthetic biology enables the production of small molecules by recombinant microbes for pharma, food, and materials applications. The secretion of products reduces the cost of separation and purification, but it is challenging to engineer due to the limited understanding of the transporter proteins' functions. Here we describe a method for genome-wide transporter disruption that, in combination with a metabolite biosensor, enables the identification of transporters impacting the production of a given target metabolite in yeast *Saccharomyces cerevisiae*. We applied the method to study the transport of xenobiotic compounds, *cis,cis*-muconic acid (CCM), protocatechuic acid (PCA), and betaxanthins. We found 22 transporters that influenced the production of CCM or PCA. The transporter of the 12-spanner drug:H(+) antiporter (DHA1) family Tpo2p was further confirmed to import CCM and PCA in *Xenopus* expression assays. We also identified three transporter proteins (Qdr1p, Qdr2p, and Apl1p) involved in betaxanthins transport. In summary, the described method enables high-throughput transporter identification for small molecules in cell factories.

## Highlights

• Transporter engineering is important in strain development.
• 361 non-essential native transporters can be disrupted via CRISPR-Cas9 in yeast *S. cerevisiae*.
• Biosensors enable high-throughput screening of transporter mutants.
• Transport of *cis,cis*-muconic (CCM), protocatechuic (PCA) acids, and betaxanthins was engineered.
• Tpo2p was validated as an importer of CCM and PCA in *Xenopus* expression assays.

## 1 Introduction

Transport of substances across biological cell membranes is essential for nutrient uptake (Davidson and Chen, 2004; McCracken and Edinger, 2013), maintenance of cellular homeostasis (Park et al., 2004; Rink and Haase, 2007), and intercellular communication (Record et al., 2014). Macromolecules are mainly transported in vesicles through exocytosis and endocytosis (Hou et al., 2012; Takei and Haucke, 2001). Small organic molecules, inorganic ions, and water are transported by transporter proteins (Yamazaki et al., 1993; Holzer et al., 2004; Abbott et al., 2009; Kell et al., 2011). Transporter malfunction may disturb cellular behavior and therefore causes many human diseases (Hediger et al., 2013; Lin et al., 2015). In industrial biotechnology, transporter engineering can result in more efficient cell factories for the production of chemicals by fermentation of renewable feedstocks (Park et al., 2014; Li et al., 2019). In an industrial process, it is preferred that the product is secreted rather than accumulated intracellularly because this relieves potential feedback inhibition and facilitates the subsequent product separation and purification (Borodina, 2019). Transporter engineering strategies altering the intracellular transport of compounds (Cardenas and Da Silva, 2016), blocking the export of the intermediate metabolites (Park et al., 2014; Li et al., 2019), or enhancing the export of the final products (Doshi et al., 2013; Hara et al., 2017; Hu et al., 2018) have been shown to improve cell factories. The engineering strategies were based on the knowledge about transporter function and specificity, which is only available for a limited number of transporters. There are several methods for transporter specificity characterization, such as phenotypic characterization of knock-out mutants (Jindal et al., 2019; Tun et al., 2014), direct activity assays in model microorganisms (Wittstock et al., 2000), oocyte cells (Nour-Eldin et al., 2006), or proteoliposomes (Bloss et al., 2002), and predictions based on sequence similarity (Li et al., 2009a; Darbani et al., 2018). The experimental methods are expertise- and resource-intensive, while the computational methods are imprecise due to the limited number of thoroughly characterized transporters. Therefore there is a need for new high-throughput methods for *in vivo* transporter characterization.

**Fig. 1:**
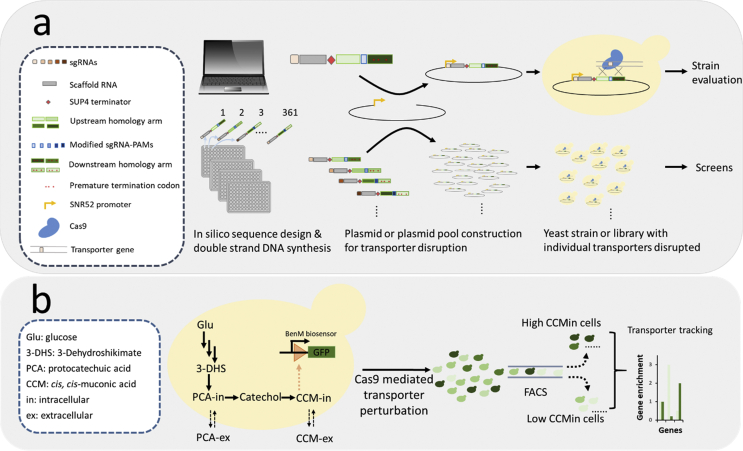
Overview of the workflow for transporter identification on the example of *cis, cis*-muconic acid. (a) Double-stranded DNA fragments were designed and synthesized for Cas9-mediated disruption of 361 *S. cerevisiae* putative non-essential transporters. The fragments comprise the sgRNA and 165 bp repair donor that introduces premature termination codons via 1 bp frameshift. (b) Detection of *in vivo cis, cis*-muconic acid (CCM) production using BenM biosensor. A transportome-wide mutant library was generated and sorted by FACS. The high- and low-fluorescent clone pools were sequenced to identify the transporters that influence CCM production.

Here we describe a method for high-throughput identification of transporters in baker's yeast *Saccharomyces cerevisiae*, a model eukaryote and industrial cell factory for fuels and chemicals (Hong and Nielsen, 2012; Nielsen, 2019). The method enables efficient disruption of the 361 non-essential native transporters via a CRISPR-Cas9 approach. The mutants can be generated individually or as transportome-wide disruption libraries (Fig. 1a). We tested the transportome-wide inactivation workflow for transporter identification on the example of xenobiotic compounds *cis, cis*-muconic acid (CCM), protocatechuic acid (PCA), and betaxanthins.

## 2 Results

### 2.1 Establishing a system for efficient transporter disruption

In yeast, 411 putative transporter genes were identified; 388 transporters as predicted by TransportDB (Darbani et al., 2018; Ren et al., 2004) and 23 transporter genes additionally listed in YTPdb (Brohee et al., 2010). Of the 411 transporter genes, 50 are essential (Zhang et al., 2004; Cherry et al., 2012) (Table S1). We aimed to establish a CRISPR-Cas9 method for the efficient disruption of transporter genes. For each of the 361 non-essential transporters, we wanted to design a plasmid harboring a gene-specific single guide RNA (sgRNA) and a repair donor that would inactivate the targeted transporter. The plasmids can be transformed, either individually or as a pool, into a Cas9-expressing yeast strain to inactivate the desired transporters. The disrupted genes in a pool of mutants can be detected by sequencing of the sgRNA-donor regions of the plasmids.

**Fig. 2:**
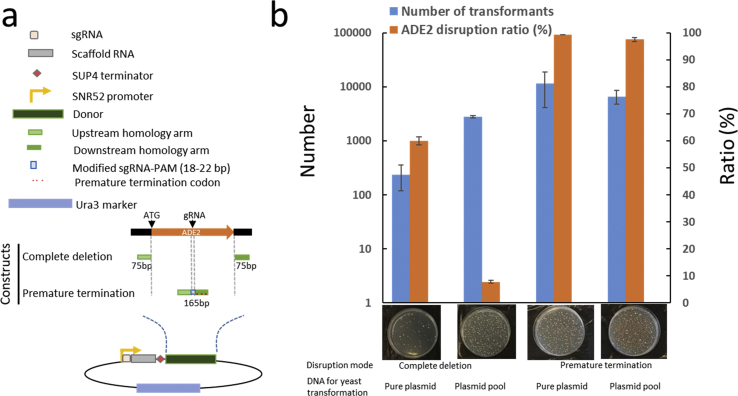
Optimization of CRISPR-Cas9-mediated gene disruption in *S. cerevisiae.* (a) Overview of the plasmid and donor fragments design. (b) The efficiency of *ADE2* disruption in a strain carrying a genome integrated Cas9 (ST8420) transformed with 1 μg of plasmid or plasmid pool. The plasmids were constructed with double-stranded DNA fragments consisting of the sgRNA-scaffold RNA-SUP4t-donor construct. Data shown are mean values ± SDs of triplicates for transformant number and *ADE2* disruption ratio, while one representative plate is shown for each transformation.

We tested two different designs of the donor fragment. *ADE2* disruption was used as a test case (Fig. 2a) because the disruption is easily detected by the red color of the *ade2Δ* yeast colonies. In one design, the donor fragment (150 bp) contained up- and down-stream sequences flanking the ORF, so the ORF would be removed completely. In the other design, the donor fragment of 165 bp was homologous to a region of the ORF and would introduce a frameshift, resulting in premature translation termination. The vector backbone and sgRNA elements were the same in the plasmids for the two designs.

To begin with, we tested pure sgRNA-donor plasmids isolated from individual *Escherichia coli* clones and verified by sequencing. For the first design with complete ORF removal, the transformation efficiency was low, only ~2·10^2^/μg plasmid (Fig. 2b). The genome editing efficiency calculated as the percentage of red colonies in relation to the total colonies was 60.0% (Fig. 2b). For the second design, the transformation efficiency was higher, above 4·10^3^/μg plasmid, and the genome editing efficiency was 99.3% (Fig. 2b). We then repeated the experiment, but this time using plasmids isolated from the whole transformation plate of *E. coli* clones (several hundred clones were obtained per plate), which would represent the way how the final library would be built. For the design with complete ORF removal, 10-fold more transformants were obtained than when using pure plasmid (~2·10^3^/μg plasmid), however, the genome editing efficiency dropped to 7.8% (Fig. 2b). For the frameshift design, the plasmid pool gave 6.6·10^3^ transformants/μg plasmid with a high genome editing efficiency of 97.5%. As the frameshift design delivered sufficient transformation efficiency and a very high genome editing efficiency, it was chosen for the creation of the transporter disruption library. We then designed sgRNAs using CRISPy (Jakociunas et al., 2015), preferentially targeting the first quarter of the ORF, for the disruption of the 361 unessential or conditionally essential transporter genes and the 165 bp donor fragments (Table S1). The sgRNA elements and the corresponding donor fragments were synthesized as individual 300 bp-double-stranded DNA and were assembled with the sgRNA vector backbone to result in the plasmid library. The library was evaluated by next-generation sequencing and PCR. It contained constructs targeting all the 361 transporters (Table S2).

### 2.2 Establishing biosensor-aided screen for *cis,cis*-muconic acid and protocatechuic acid in *S. cerevisiae*

To enable high-throughput screening of the yeast strain libraries with disrupted transporters, we designed a biosensor-aided screen for the target xenobiotic organic acids CCM and PCA. The biosensor constructs for CCM and PCA consisted of constitutively expressed transcriptional regulators (BenM variant MP02_D04 (Snoek et al., 2020) or VanR-PcaQ (Skjoedt et al., 2016; Gitzinger et al., 2012), respectively) and a reporter GFP expression cassette under the control of a promoter with the binding sites for the transcriptional regulators (Skjoedt et al., 2016; Ambri et al., 2020).

**Fig. 3:**
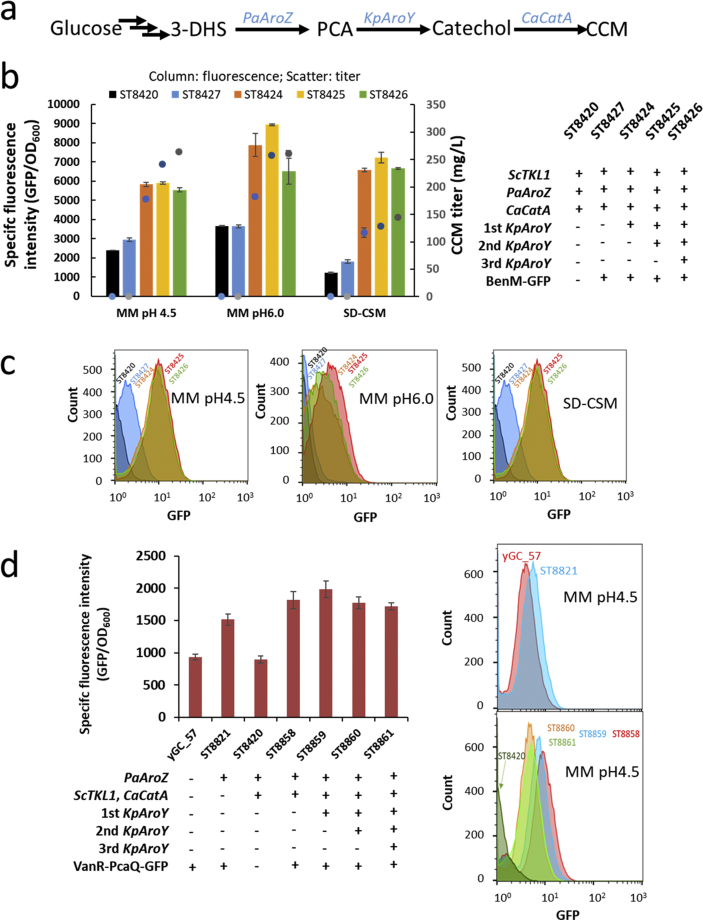
Biosensor setup and validation for the high-throughput screen. (a) The metabolic pathway for PCA and CCM biosynthesis. (b) Fluorescence output of CCM-responsive BenM biosensor in CCM producing strains. (c) Flow cytometric analysis of the CCM producing strains harboring BenM biosensor. (d) Fluorescence output of PCA-responsive PcaQ biosensor in PCA producing strains. MM, mineral medium, SD-CSM, synthetic defined medium with complete supplement mixture. Data shown in panel b and d are mean values ± SDs of biological triplicates, while a representative sample is shown for flow cytometric analysis in panel c and d.

First, we sought to select a suitable parental strain and a cultivation medium that would allow the resolution of different metabolite production levels. The different production levels of CCM and PCA were achieved by integrating one, two, or three copies of PCA decarboxylase from *Klebsiella pneumonia* into a strain (ST8420) expressing the other genes completing the CCM biosynthetic pathway, 3-dehydroshikimate dehydratase from *Podospora anserina* (PaAroZ) and catechol 1, 2-dioxygenase from *Candida albicans* (CaCatA) (Fig. 3a and S1). The three strains with one, two, or three copies of PCA decarboxylase resulted in 131, 163, and 178 mg/L CCM respectively, with concurrent PCA titers of 260, 161, and 119 mg/L (Fig. S1) on mineral medium pH 6.0. The more copies of PCA decarboxylase were present, the higher the titer of CCM and the lower the titer of PCA.

We introduced the BenM variant MP02_D04 (Snoek et al., 2020) into the three strains, to test the fluorescence intensity. We selected mineral medium (MM) with different pH values (pH 4.5 or pH 6.0) and synthetic defined (SD) medium with default pH ~5.2. pH value of the medium influences the transport of weak organic acids (Abbott et al., 2009). The resulting strains showed consistently increased extracellular CCM titer on all three media (ST8424 < ST8425 < ST8426, Fig. 3b), and both ST8425 and ST8426 had a higher intracellular CCM compared to ST8424 (Fig. S2). However, only the difference in the CCM between ST8424 and ST8425 strains could be detected by the fluorescence response, when the cells were cultivated in mineral medium at pH 6.0 or synthetic defined medium (Fig. 3b and c). The lack of correlation may be attributed to the limited linear detection range of the BenM sensor, which is influenced by a set of factors including pH (Fig. 3b and c and Table S3) that affects the protonation and transport of CCM (Snoek et al., 2018). The strain ST8424 was thus selected as the parental strain for creating and testing the transporter disruption library for CCM transporters. The tests were designed to be carried out in mineral medium at pH 6.0, the conditions that gave the best resolution of the fluorescence signal.

In a similar approach, we validated the VanR-PcaQ mediated response to PCA. We introduced *PaAroZ* into strain yGC_57 equipped with an optimized biosensor design, where GFP expression was repressed by VanR (Gitzinger et al., 2012) but could be transcriptionally activated by PcaQ when PCA was present (Skjoedt et al., 2016). Using this PCA biosensor, we could only verify an increase in fluorescence as a response to the PCA production when the strains were grown on mineral medium (MM) pH 4.5 or MM pH 6.0, but not on YPD or synthetic defined media (Fig. S3). We further evaluated the ability of the PcaQ biosensor to detect different levels of PCA. We implemented the PcaQ biosensor into three PCA producing strains that had decreasing PCA producing capabilities (Fig. S1), to generate ST8859, ST8860, and ST8861 (Fig. 3d). We observed a reduction in fluorescence corresponding to decreased PCA levels in ST8859 and ST8860 (Fig. 3d), but not in ST8860 and ST8861. We, therefore, selected ST8859 as the parental strain for the transporter disruptions and mineral medium at pH 4.5 as cultivation medium.

### 2.3 Establishing a transporter disruption library and screening for transporters with influence on PCA and CCM levels

The constructed plasmid library was transformed into the optimized parental strains, ST8424, and ST8859, to generate the yeast libraries towards CCM (Yealib-CCM) and PCA (Yealib-PCA) transporters, with a 714- and 311-fold coverage achieved, respectively, for 361 individual transporters.

The fluorescence intensity from the integrated biosensors was analyzed, and four different cell populations were enriched by two rounds of fluorescence activated cell sorting (FACS): either the highest (CCM-Max, PCA-Max) or the lowest (CCM-Min, PCA-Min) single-cell fluorescence intensity. From these libraries, 40,000 cells were collected for each population in each round. The identified populations, when tested as a pool, did not produce more extracellular CCM (Fig. S4a) or PCA (Fig. S4b) than control strain, but this could be because the mutants with significant changes of CCM or PCA titers were masked by other lower producing mutants.

To identify the transporter disruptions responsible for the change in CCM and PCA production, we analyzed the enriched transporter disruptions by Nanopore sequencing. Out of a total of 361 target genes, 358 and 357 were detected in the unsorted transporter disruption yeast libraries, Yealib-CCM (CCM-Con, Table S4), and Yealib-PCA (PCA-Con, Table S5), respectively. The sequencing of the CCM-Min, CCM-Max, PCA-Min, and PCA-Max identified 344, 350, 270, and 349 genes, respectively (Tables S4 and S5). We did not find enrichment in specific classes of transporters with similar function/functional mechanisms (Fig. S5), but we observed an enrichment in a set of individual transporters (Figs. S6 and S7). The highest enriched transporter disruptions were selected based on enrichment scores in both Max and Min groups and read analysis through differentially expressed genes algorithm (Fig. S8). A total of 24 genes, 9, 6, 3, and 6 genes, enriched in CCM-Min, CCM-Max, PCA-Min and PCA-Max (Figs. S8, 4a, and 4b), respectively, were chosen for further validation.

### 2.4 Reverse engineering of transporter disruptions

**Fig. 4:**
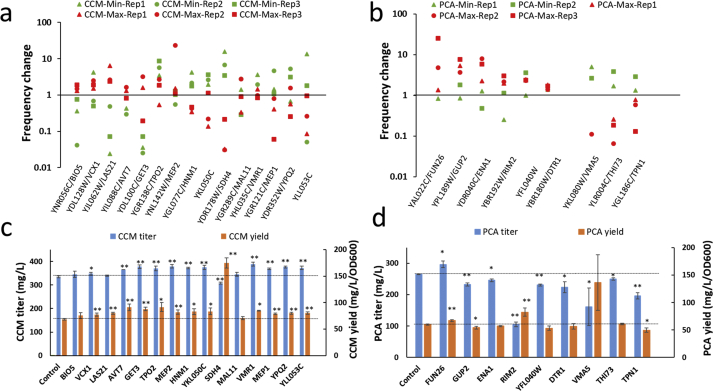
Frequency change-based transporter identification for altered CCM and PCA production. (a, b) Frequency change of transporter genes for reverse validation. The frequency of each transporter was defined as the ratio of the read count to the total experimental counts for each sample. Frequency change for each gene was compared with the corresponding control samples. (c, d) The CCM or PCA production of strains reverse engineered with the transporter disruption. ST8424 and ST8859 were used as the parental strain, respectively, and metabolite was quantified on 72 h culture. Data shown are from biological replicates in panel a and b, and data shown in panel c and d are mean values ± SDs of biological triplicates. Statistical differences between the control and test strains were determined by two-tailed Student's *t*-test. *P < 0.05; **P < 0.01.

To validate the effect of the candidate transporters, we constructed plasmids for disruption of the individual transporters, in the host strains ST8424 or ST8859, carrying the complete biosynthetic pathway for PCA and CCM. To minimize any bias resulting from differences in growth, we compared both titers (in mg/L) and specific yields (in mg/L/OD_600_) (Fig. 4c and d). Disruption of transporters selected from the same enrichment group did not result in a consistent increase or decrease of the extracellular product titer. The biosensor signal is dependent on the intracellular concentrations of CCM/PCA, which may correlate differently with the extracellular concentration, depending on the nature of the transporters. Thus, the biosensor screening approach is somewhat indirect. While it detects the intracellular changes of the metabolites of interest, the direction in which the extracellular product concentration changes must be experimentally tested. Disruption of any of *VCX1*, *LAS21*, *AVT7*, *GET3*, *TPO2*, *MEP2*, *HNM1*, *YKL050C*, *SDH4*, *VMR1*, *MEP1*, *YPQ2,* or *YLL053C* genes in ST8424 improved CCM specific yield, with the biggest improvements, 33% and 155%, achieved by *TPO2* and *SDH4* disruptions respectively (Fig. 4c). Furthermore, *TPO2* was identified as the only transporter gene that, upon disruption, gave a 20% improvement in PCA yield, while *SDH4*, *MEP1*, *YPQ2,* and *YLL053C* disruptions reduced the PCA yield by up to 14% (*YPQ2*) (Fig. S9a). Disruption of *FUN26* and *RIM2* in ST8859 increased the PCA yield by 11% and 37%, respectively, while *GUP2* and *TPN1* disruption reduced it by 10% and 17% (Fig. 4d). The CCM yield in ST8859 was enhanced by *GUP2*, *YFL040W,* and *THI73* disruptions by 11%, 12%, and 19%, respectively (Fig. S9b). It is noted that *SDH4*, *RIM2*, and *VMA5* disruptions resulted in lower titers and yields on glucose, but in higher specific yields. One possible reason for such phenotype in *SDH4* disrupted strain is that the impaired succinate dehydrogenase resulted in overflow metabolism (Lemire and Oyedotun, 2002; Raab et al., 2010; Chang et al., 2015), which could also support an elevated flux to the shikimate pathway. These three genes were not considered for further study due to the severely retarded biomass accumulation and lower product titers.

To test for combinatorial effects, we grouped the 8 identified transporter genes into three groups. Group I consisted of transporter disruptions that resulted in increased CCM but decreased PCA yield (*VMR1*, *YPQ2*, *GUP2*). Group II consisted of transporter disruptions resulting in increased CCM and PCA yield (*AVT7*, *TPO2*), and group III consisted of transporter disruptions resulting in increased CCM yield, while PCA yield remained unchanged (*GET3*, *MEP2*, *YKL050C*). We then generated strains with combinations of transporter disruption from the three groups. All the combinations failed to give a further improvement of the CCM production compared to the single disruptions (Fig. S10). It may result from the transporters’ indirect effects, which could perturb cellular metabolism and homeostasis, and cell growth (Fig. 4c and d).

*TPO2* disruption resulted in the most significant improvement and didn't retard the cell growth. Tpo2p is a member of the polyamine transporter family, and we investigated whether the other members of the polyamine transporter family also had an impact on CCM and PCA production. We disrupted *TPO1*, *TPO4*, or *TPO5* in strain ST8424. *TPO1* disruption resulted in an increase in the CCM yield by 11%, while it did not affect the PCA yield (Fig. S11). In contrast, *TPO4* and *TPO5* disruptions did not affect either CCM or PCA yield (Fig. S11).

### 2.5 Transporter activity of Tpo2p on CCM and PCA

**Fig. 5:**
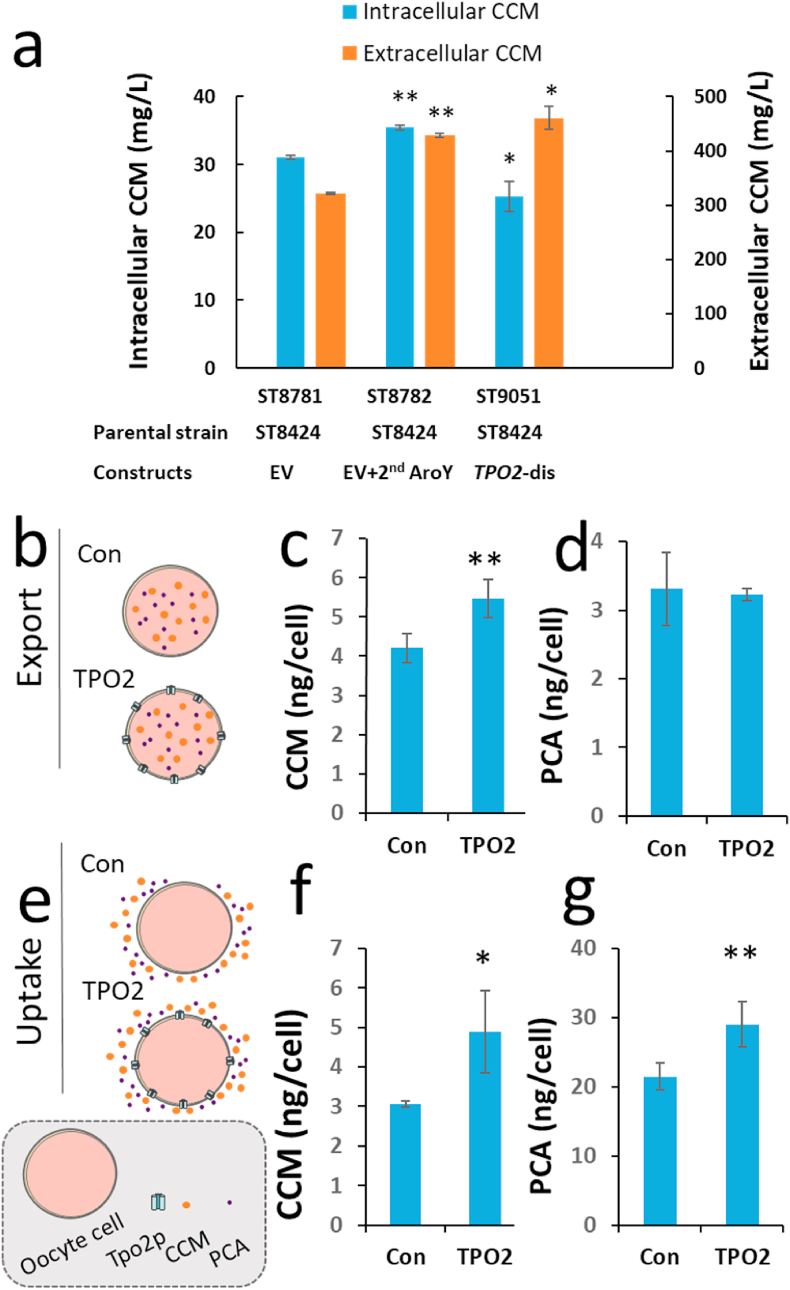
Characterization of transport activity of Tpo2p. (a) Intracellular and extracellular concentrations of CCM in the yeast strains with disrupted Tpo2p. EV – ST8424 with an empty vector (negative control). EV+2^nd^ AroY - ST8424 expressing an additional copy of *AroY* (positive control). *TPO2*-dis – ST8424 with a disrupted *TPO2* gene. The strains were cultivated for 72 h in MM pH 6.0. (b–g) The transporter activity of Tpo2p on CCM and PCA. Oocyte cells expressing *TPO2* were injected with CCM and PCA solution (b, c, and d) or immersed into CCM and PCA solution (e, f, and g). Cellular CCM and PCA were extracted and measured using LC-MS. Data shown are mean values ± SDs of biological triplicates. Statistical difference between control (Con) and indicated samples was determined by two-tailed Student's *t*-test. *P < 0.05; **P < 0.01.

Tpo2p is a polyamine transporter of the 12-spanner drug:H(+) antiporter DHA1 family, previously reported to increase the spermine uptake and vacuolar spermine concentration when overexpressed (Tomitori et al., 2001). There are no reports on its activity towards CCM or PCA. We found that the intracellular concentration of CCM was 19% lower in the *tpo2Δ S. cerevisiae* mutant in comparison to the control strain producing CCM (Fig. 5a), indicating that Tpo2p imports CCM. To validate whether indeed Tpo2p can transport CCM and PCA, we performed characterization of the transporter in *Xenopus* oocytes, a robust system for transporter characterization. Even though oocytes have low background transport activity and only express few endogenous membrane proteins (Jørgensen et al., 2017), they still showed some background CCM and PCA transport activity (Con in Fig. 5b–g). Nevertheless, in the export assays, the oocytes expressing Tpo2p retained 30% more CCM intracellularly than control oocytes (Fig. 5b and c). In the uptake assays, the Top2p-expressing oocytes accumulated 60% more CCM than controls (Fig. 5e and f). While oocyte expressing *TPO2* showed no difference in the intracellular PCA amount in the export assay (Fig. 5b and d), they accumulated 35% more PCA in the uptake assay (Fig. 5e and g). These experiments confirm that Tpo2p functions as CCM and PCA importer.

### 2.6 Transporter identification for betaxanthins

**Fig. 6:**
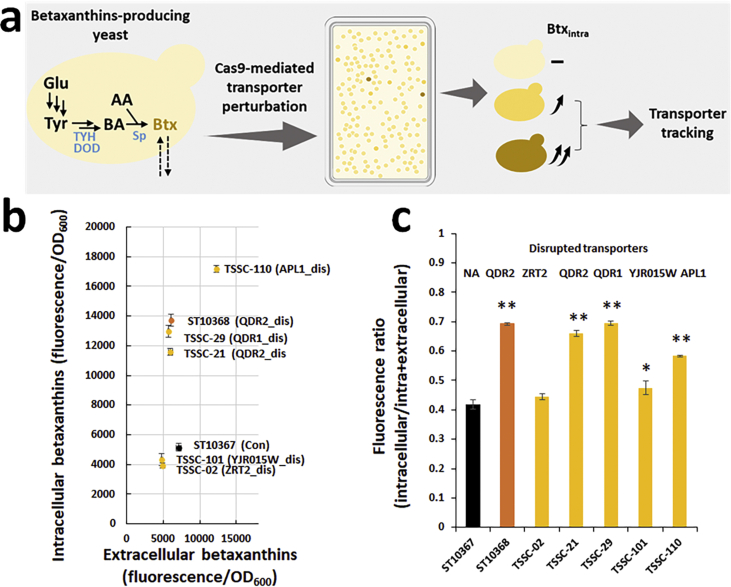
Transporter identification for betaxanthins (a) Tyrosine hydroxylase (TYH) and DOPA dioxygenase (DOD) were expressed in yeast to produce betalamic acid (BA). BA spontaneously reacts with intracellular amino acids, generating yellow to orange fluorescent pigments - betaxanthins. The betaxanthins producing yeast is transformed with plasmid library for transporter disruption, and the resulting clones are tested for increased intracellular betaxanthins accumulation (Btx_intra_). The causal transporter disruption are identified by sgRNA-donor sequencing. Betaxanthins production (b) and distribution (c) by variant strains pre-selected in the screening, based on increased color intensity in colonies and higher cellular betaxanthins retention. Data shown in panel b and c are mean values ± SDs of biological triplicates. Statistical difference between control (ST10367) and indicated strains was determined by two-tailed Student's *t*-test. *P < 0.05; **P < 0.01.

We further applied the high-throughput transporter identification method to betaxanthins, which are yellow to orange fluorescent pigments made by spontaneous conjugation of betalamic acid with intracellular amino acids. The pathway for betalamic acid production is present in plants and consists of two enzymes, tyrosine hydroxylase (TYH) and L-DOPA dioxygenase (DOD) (Gandia-Herrero and Garcia-Carmona, 2013). We expressed TYH from *Beta vulgaris* (*BvCYP76AD1*^W13L, F309L^) and DOD from *Mirabilis japonica* (*MjDOD*) in *S. cerevisiae* CEN.PK strain (Fig. 6a). The strain retained 79–81% of the total betaxanthins in the cell (Figs. S12 and S13). The strain was transformed with plasmid library for transporter disruption, and the colonies were plated on mineral medium and incubated for 4 days. Eighty colonies that visually had a more intense yellow color were selected for further evaluation. The eighty strains were cultivated in a liquid synthetic defined medium, and the intracellular and extracellular fluorescence was measured on spectrophotometer (Fig. S12), in one replicate. The eight strains, which showed the highest cellular betaxanthins fluorescence in the first-round evaluation, were further validated in three biological replicates, and six showed slightly higher cellular betaxanthins retention (86–87% vs. 81% in control strain) (Fig. S13). The transporter disruptions in the six selected strains were identified by sequencing. Four of the six strains carried disruptions of *OXA1*, and two remaining strains had disruptions of *MRS2*. Oxa1p is a mitochondrial inner membrane insertase that mediates insertion of proteins into the mitochondrial membrane. Mrs2p is a mitochondrial inner membrane Mg(2+) channel. The mechanism by which the deletion of these two proteins influences betaxanthins cellular retention is unknown.

In CEN.PK strains, betaxanthins were mostly retained intracellularly (ca. 80%), and this complicated the screening for exporters using our transporter disruption library. We, therefore, investigated if the native export of betaxanthins would be higher in another strain background S288c. We expressed TYH and DOD in S288c strain, and, surprisingly, only ~40% of betaxanthins were intracellular. As further verification, we disrupted *QDR2* encoding a quinidine/cations-related transporter previously reported to impact betaxanthins distribution (Savitskaya et al., 2019) and found that the fraction of intracellular betaxanthins increased from 40 to 66% (Fig. S14). We then transformed the S288c background strain, carrying both betaxanthins pathway and Cas9, with transporter disruption library and screened the resulting transformants on mineral medium supplemented with histidine, leucine, and methionine, after incubation for 3–4 days. By color intensity-based screening of ca. 23,000 variant strains, we selected 81 strains with increased yellow color. These strains were cultivated in liquid synthetic defined medium and intracellular and extracellular betaxanthins were analyzed by measuring fluorescence on spectrophotometer, in one replicate. 31 strains retained more than 50% of betaxanthins intracellularly (Fig. S14). To identify transporters with more diverse impacts, we grouped these strains into three groups according to the ranges of cellular betaxanthins fraction: 50–60%, 60–70%, and > 70% (Fig. S14b). We then randomly selected three strains from each group, sequenced the sgRNA-donor in the nine strains for transporter verification (Fig. S14b), and validated the betaxanthins distribution of the variant strains in three biological replicates (Fig. 6b and c). Five of the nine strains carried *QDR2* disruption, and the remaining four strains harbored disruption of either *QDR1*, *APL1*, *YJR015W*, or *ZRT2* (Fig. S14b). The disruption of *QDR2*, *QDR1*, and *APL1* all increased the intracellular betaxanthins amount (> 120%, Fig. 6b) and the cellular betaxanthins fraction (58–70% vs. 42%, Fig. 6c). Qdr1p and Qdr2p are multidrug transporters of the 12-spanner drug:H(+) antiporter DHA1 family. Their deletion likely retards the export of betaxanthins or their precursors out of the cell. Apl1p is a subunit in the clathrin-associated protein complex (AP-2), which is involved in vesicle transport. Of these three transporter targets that influence betaxanthins production, only one (*QDR2*) was previously identified in a screen of genome-wide deletions conducted on betaxanthins-producing strain (Savitskaya et al., 2019).

## 3 Discussion

Here we demonstrate a method for efficient CRISPR-Cas9 mediated transporter disruption and apply this to rapidly characterize transporters with activity towards the endogenously-produced xenobiotic compounds CCM, PCA, and betaxanthins. In this process, we implement a biosensor-aided screen and Nanopore sequencing-guided reverse validation.

The use of a simple plasmid transformation for CRISPR-Cas9 mediated gene disruption was adapted to generate a library of engineered *S. cerevisiae* strains (Roy et al., 2018). This approach is more convenient than a deletion library (Kitagawa et al., 2011), as it circumvents the need for integrating multiple metabolic pathway genes into different knock-out strains individually. The transporter disruption via 1 bp frameshift instead of full deletion of the coding sequence (Jessop-Fabre et al., 2016) enabled efficient manipulation of 361 unessential and conditionally essential *S. cerevisiae* transporters, creating a yeast cell library for screening (97.5% of transformants are positive) or individual strains for evaluation (99.2–99.4%). Disrupted transporters were not evenly distributed in the yeast cell libraries, with the read number for individual transporters for Yealib-CCM varying from 2 (YKR103W) to 7722 (YCR010C). This is likely due to the differences in growth rates of the disrupted strains and differences in sgRNA efficiencies. However, the library can be screened with a colony based method (for example, color intensity) to minimize the growth impact. When the library propagation is essential, the library, as of its small size, can be thoroughly analyzed by increasing the screening coverage (>100-fold in this work), which is feasible for medium or high-throughput screening, such as FACS. BenM and VanR-PcaQ biosensors aided FACS was employed in the identification of single cells with a change in fluorescence output caused by the altered transport of CCM or PCA. The process optimization for the use of BenM and VanR-PcaQ (Fig. 2, S2 and S3), including the strain and cultivation processes, allowed the fluorescence change to be detected. In order to further minimize false positive rate, we have set up stringent criteria for FACS screening and performed two successive selection rounds. Nearly all transporter targets selected from the FACS screening resulted in a significant effect on the product titer as confirmed by reverse engineering.

The plasmids used in this work contained both sgRNA and donor, as originally proposed by Garst et al. (2017). This enables easy tracking of the disrupted transporter and aids in efficient identification of the causal transporter disruptions. In this work, we based the identification of the causal transporter disruption on (Nanopore) sequencing of the sgRNA-donor (Manrao et al., 2012). From the sequencing data, enrichment score is obtained for each transporter, which guides the analysis for the selection of potentially effective transporters. This setup realized with high accuracy the identification of transporters relevant for CCM, PCA, and betaxanthins (13 experimentally verified functional transporters out of 15 selected candidates for CCM (Fig. 4c), 4 out of 9 for PCA (Fig. 4d), 4 out of 5 for betaxanthins (Fig. 6c)). Such a workflow enables robust identification of functional transporters.

Via experimental validation, 19 and 13 potential transporters were identified affecting the production of CCM and PCA, respectively (Fig. 4 and S9). The small change in CCM/PCA production upon transporter disruption (Fig. 4 and S9) is likely because of the functional redundancy of the transporters. When the transport of a compound is mediated by multiple transporters, the disruption of one transporter may only give a small change or even no effect due to the complementary action of the other transporters (Wang et al., 2020a). It may also account for that *TPO2* disruption was not found in PCA transporter screening, where the change of fluorescence upon the disruption of *TPO2* was not large enough to be captured. The large number of the identified transporters indicates the complex complementary effects of multiple transporters for CCM and PCA. A similar observation has been made for the hexose and lactate transport, where hexose transport was abolished only when 20 responsible transporters were deleted (Wieczorke et al., 1999), and the deletion of 25 potential transporters didn't change the lactate production rate (Mans et al., 2017). The transportome represents a complex interaction network, where the expression of various transporters is co-regulated (Godinho et al., 2017; Hauser et al., 2001) while the degradation of some transporters is associated with the cell state (Souza et al., 2011; Umebayashi and Nakano, 2003). The transportome complexity is also highlighted by the strain background-dependent impact, where *QDR2* disruption increased cellular betaxanthins fraction only in S288c background strain (Fig. S14) but not in CEN.PK background strain (Fig. S12). The difference is possibly due to the lower *QDR2* expression level in CEN.PK background strain (ca. 75% lower (Espinosa et al., 2019)). Furthermore, the identified transporters are located in both plasma membrane and in the membranes of organelles, such as mitochondria and vacuole. These transporters, besides the direct transport activity (Fig. 5), may also indirectly affect the xenobiotic compound transport and production by perturbing the cellular metabolism and homeostasis (Olin-Sandoval et al., 2019). With the identified transporters as the starting point, further exploration of their additional roles may help to construct more efficient cell factories.

The validated transporters for the two xenobiotic organic acids CCM and PCA (Fig. 4 and S9) are involved in the transport processes of various native chemicals. Moreover, we found that the transport activity towards CCM or PCA is not common for a specific family of transporters (Fig. 4, Tables S4 and S5), but is specific for individual transporter proteins. Despite this, the *TPO2* and *TPO1* disruption increased the PCA (Fig. S9a) and *p*-coumaric acid (a previously studied tyrosine-derived aromatic metabolite) (Rodriguez et al., 2017), respectively, and the disruption of both transporters (Fig. 4 and S11) and *QDR3* overexpression (Pereira et al., 2019) enhanced the CCM production. The *QDR1* and *QDR2* disruption increased the cellular betaxanthins retention (Fig. 6). These impacts, regarding the production of the aromatic compounds, suggest a potential for engineering these five transporters, related to spermine and quinidine, for improving the production of shikimate pathway-derived compounds.

The presented method can be applied for the investigation of transporter effects on the production of various chemicals and also on the uptake of various substrates. As the library size is small, it should be possible to apply the method even if no high-throughput screening method is available. It is, however, most convenient if a high-throughput method can be implemented via biosensor or growth-coupling. As many transport processes are reversible, potentially uptake assays that are based on growth and are, therefore, very high-throughput could also be applied to investigate the transport of metabolic products.

In summary, we established a high-throughput method for transporter characterization for xenobiotic compounds in yeast *S. cerevisiae*. The transportome complexity was explored, and transporter targets for improved CCM, PCA, and betaxanthins production were identified. These beneficial targets directly or indirectly affecting compound transport expand our understanding of the transporter functions.

## 4 Materials and methods

### 4.1 Strains and plasmids

*E. coli* DH5α was used as the host for the construction of plasmid (library) and its propagation. Yeast strains (Table S6) derived from CEN.PK 102-5B (Entian and Kotter, 2007) were used throughout this study. The yeast strain construction was performed using a CRISPR-Cas9 system (Jessop-Fabre et al., 2016). For the construction of yeast cell library or strains with individual transporters disrupted, a plasmid construct with both sgRNA cassettes and the donor fragments for full knock out or premature termination introduction was used to enable the gene disruption using the transformation of a single plasmid. For the gene integration or multiple transporter disruption, the donor DNA fragments at a high amount (approx. 500 ng each) were employed for efficient strain construction.

Plasmids (Table S7) were constructed using the Gibson assembly with Gibson Assembly® Master Mix (New England BioLabs) or the EasyClone method (Jensen et al., 2014). Synthesized DNA fragments were utilized for *ADE2* disruption optimization (Genscript or Twist Bioscience, Table S8) and for single transporter disruption or construction of plasmid pool for yeast cell library with transporter disruption (Twist Bioscience) via Gibson assembly. Donor DNA fragments for genome integration or multiple transporter disruptions were constructed by overlap PCR (Zhou et al., 2012) or PCR, respectively, with primers listed in Table S9.

Yeast transformation was performed following the standard lithium acetate method (Gietz and Woods, 2002). Yeast transformants were subjected to additional 2 h outgrowth on synthetic defined (SD) medium with complete supplement mixture before plating onto selective plates for transporter disruption and gene integration. Transformants were validated by either genomic PCR verification (gene integration) or sequencing of the genomic PCR product (gene disruption).

### 4.2 Biosensors designs

BenM variant MP02_D04 was under the control of a weak Rev1 promoter. The truncated 209bp-CYC1 reporter promoter driving the expression of GFP was engineered to remove the upstream activating sequences and carry the BenM operator site (Skjoedt et al., 2016).

The PCA sensor PcaQ is cloned under the control of the strong TDH3 promoter while the engineered TEF1 reporter promoter carries both the PcaQ operator site (PcaO; GATCGTATAACCTCCTGGTTAAGGGAAAGCCACGAAATATCATTTTACCTAACCGGATGAAACATCCAAATCTGACGACG) and two VanR operator sequences separated by the Eco47III restriction site (VanO; ATTGGATCCAATagcgctATTGGATCCAAT) (Ambri et al., 2020). The PcaO operator sequence is placed 265 bp upstream of the TATA element while VanO is positioned 47 bp downstream of the TATA element. VanO was introduced to lower the OFF state of the reporter promoter as previous reports suggest that lowering the basal expression of the reporter promoter is preferred when employing prokaryotic transcriptional activators in yeast (Skjoedt et al., 2016).

### 4.3 Medium and strain cultivation

*E. coli* strains harboring plasmid were cultivated at 37 °C on Luria-Bertani (LB) medium with 100 mg/L ampicillin. Yeast strains were routinely maintained on YPD (10 g/L yeast extract, 20 g/L peptone, 20 g/L glucose) or selective plates (for engineered strains containing plasmids). Yeast transformants were selected on either YPD with 100 mg/L nourseothricin, YPD with 200 mg/L G418 or SD-Ura (20 g/L glucose, 6.7 g/L yeast nitrogen base, complete supplement mixture lacking uracil), mineral medium (MM) (Jensen et al., 2014), MM supplemented with 100 mg/L leucine, 20 mg/L histidine, and 20 mg/L methionine (MM + Leu + His + Met) plates.

Yeast cells were cultivated in liquid media for strain characterization and product quantification, including YPD, MM (pH 6.0 or 4.5) without or with supplementation of 20 mg/L uracil or 20 mg/L histidine, SD (20 g/L glucose, 6.7 g/L yeast nitrogen base), SD-Ura, SD-His (20 g/L glucose, 6.7 g/L yeast nitrogen base, complete supplement mixture lacking histidine), and SD-CSM (20 g/L glucose, 6.7 g/L yeast nitrogen base, complete supplement mixture).

Yeast strains were all cultured at 30 °C in this study, in 96-deep well plate at 300 rpm in a New Brunswick™ Innova® 44 shaker or MTP-48-Flowerplate at 1000 rpm in microbioreactor system BioLector (m2p-labs). Overnight preculture of fresh single colonies were inoculated into 600 μL (96-deep well plate) or 1 mL (MTP-48-Flowerplate) of the corresponding medium with the initial OD_600_ as 0.05 for the sub-cultivation.

### 4.4 Optical density and fluorescence measurements

Optical density at 600 nm, OD_600_, indicating cell density, GFP fluorescence (λ_Excitation_ 485 nm, λ_Emission_ 515 nm) of the biosensors' output, and betaxanthins fluorescence (λ_Excitation_ 485 nm, λ_Emission_ 525 nm) was measured with a 200 μL yeast culture diluted 2–10 fold on a microtiter plate reader BioTek Synergy MX (BioTek). The fluorescence measurement was performed using 96-well clear-bottom black plates (Corning), with the diluted medium as a blank. Specific fluorescence, the normalized fluorescence data obtained by dividing the measured fluorescence value by the measured OD_600_, was shown.

The GFP fluorescence value of PcaQ biosensor output measured by Biolector was collected with a gain value of 100.

### 4.5 Flow cytometric analysis

Single-cell fluorescence of 24 h yeast subcultures was analyzed with flow cytometry using the MACSQuant VYB (Miltenyi). With the yeast culture diluted in 10-time with water in 96-well microtiter plate, GFP fluorescence from the biosensor was measured using the B1 channel (excitation 488 nm and emission 525/50 nm). Data was collected on 30,000 cells for each sample and processed using the FlowJo software.

### 4.6 Metabolite quantification

The CCM and PCA produced by yeast cells were quantified with HPLC following the procedures described in the previous report (Skjoedt et al., 2016; Wang et al., 2020b). Briefly, cell culture was diluted 5- or 10-time with water and centrifuged at 4000 rpm for 5min, and the supernatant was then used for the extracellular metabolite analysis. Since CCM and PCA were not degraded by *S. cerevisiae*, the intracellular metabolite was extracted from water-washed cells of 500 μL sub-culture by vortexing the cells in 400 μL water at 95 °C for 1 h. The intracellular and extracellular metabolite samples were analyzed using HPLC with Aminex HPX-87H ion exclusion column kept at 60 °C, and the CCM and PCA were detected by a UV detector (Dionex) at 250 and 220 nm respectively. The analysis was run with an injection volume of 20 μL and with eluent 1 mM H_2_SO_4_ at a flow of 0.6 mL/min for 45 min. The compound concentrations were quantified by comparison with the spectrum of the reference standards and based on the standard calibration curve for 0–100 mg/L CCM and PCA.

### 4.7 Library construction

DNA fragments consisting of sgRNA-scaffold RNA-SUP4t-donor (300 bp, Table S1) and the end adaptors for synthesis (43 bp) (Twist Bioscience) were individually synthesized first for 361 transporters, including unessential transporters and conditional essential transporters. All sgRNAs for CEN.PK background yeast strain were designed with the web-based tool CRISPy (Jakociunas et al., 2015). Specifically, sgRNA with ≤ 3 nucleotide repeat, ≤ 50% GC contents, minimum mismatch, and located in the first ¼ part of the transporter coding sequence, was preferentially chosen from the candidate list. It is to enable an efficient guiding of the Cas9 and minimize the interference of post-translational processes by the truncated transporters. The donor sequence (165 bp) was generated by changing the sgRNA-PAM sequence (23 bp) to a different sequence with 22 bp in length to introduce the premature termination codons for transporter disruptions.

The plasmid library, consisting of 361 plasmids for individual transporter disruptions, was constructed using Gibson assembly of PCR amplified backbone (pQC003) and the pooled synthesized DNA fragments. A total of five smaller libraries were first built to enable the use of the transporter disruption library for multiple projects, also where the high-throughput screen is not available. In these cases, smaller libraries can be investigated using low-throughput HPLC, mass spectrometry, or similar methods. The five libraries contained transporters grouped as following: (i) 73 ATP dependent transporters (AD-1 to AD-5 (Table S1)), (ii) 79 secondary transporters (SeTS-1 to SeTS-5 (Table S1)), (iii) 65 secondary transporters (SeTS-6 to SeTS-9 (Table S1)), (iv) 84 secondary transporters (SeTS-10 to SeTS-14 (Table S1)) and (v) 60 other transporters, including ion channel transporters, unclassified transporters and transporters that were only annotated in YTPdb, but not in TransporterDB (Table S1).

The five subgroups of fragments were assembled with the PCR amplified backbone to result in five plasmid pools. To ensure that each transporter disruption construct was well represented in the library, the fragments were mixed in equimolar ratios for the Gibson assembly. After the *E. coli* transformation, the plasmid library/pool was then extracted from the scraped cells of the 16 h transformants on LB plates containing 100 mg/L ampicillin. The five sub-libraries, Sctrans-PP-Grp1, Sctrans-PP-Grp2, Sctrans-PP-Grp3, Sctrans-PP-Grp4, and Sctrans-PP-Grp5, were mixed to generate the plasmid library (Sctrans-PP) with individual plasmids in equal molar concentrations.

These plasmid libraries were verified through next generation sequencing of amplicon, and subsequent data analysis, following the procedures described before (Wang et al., 2019). Briefly, the sgRNA-donor region for the six individual plasmid libraries was prepared by PCR amplification using primer sets Ampl-5adap-F/Ampl-3adap-R, and subjected to a second index PCR. The resulting amplicons were purified and pooled for the sequencing on the Miseq (Illumina) platform with the paired-end reads protocol and read lengths of 2 x 250 nt. > 240,000 reads were collected. The read count for each transporter was analyzed as described in the subsequent ‘Sequence data processing’ section, and listed in Table S2. The plasmids targeting three genes, YPR124W, YKL064, and YIL170W had no reads mapped. The existence of these three plasmids was verified through PCR (Table S2) using specific primer sets (Table S9). The plasmid libraries can be obtained from Addgene (accession numbers 153101 - 153106).

Yealib-CCM and Yealib-PCA were constructed by transforming the plasmid library, Sctrans-PP, into a parental strain, ST8424 or ST8859, following the optimal procedures described above.

### 4.8 Yeast cell screens for CCM and PCA transporters

After two-day growth on the SD-Ura plates at 30 °C, yeast transformants for Yealib-CCM and Yealib-PCA were collected by scraping and resuspension in fresh MM pH 6.0 and MM pH 4.5 respectively. The cell suspension was then inoculated into 600 μL MM pH 6.0 (Yealib-CCM) or MM pH 4.5 (Yealib-PCA) accordingly in 96-deep well plate, with an initial OD_600_ of 0.05. The cells were cultivated for 24 h, and the single-cell fluorescence of the culture analyzed using the BD Biosciences Aria II (Becton Dickinson) with a blue laser (488 nm). The single cells in the cell population were first defined according to the distribution in forward cell scatter (FSC), and the Yealib-CCM and Yealib-PCA cells with the highest and lowest fluorescence outputs were gated based on the FITC response difference between the samples and their corresponding control samples. 40,000 cells were sorted for each group, and the sorted cells from the 1^st^ round screening were cultured on MM for 36 h for glycerol stock preparation.

For the 2^nd^ round screening, the sorted cells from the glycerol stock were inoculated into the MM pH 6.0 or MM pH 4.5, respectively, for pre-cultivation. The resulting preculture was then inoculated into 600 μL MM pH 6.0 or MM pH 4.5, with the initial OD600 as 0.05 for 24 h cultivation. The cell analysis and sorting and glycerol stock preparation for the 2^nd^ round screening was performed following the procedures described above.

For the metabolite production quantification of the sorted cells, cells from glycerol stocks of each sample were inoculated into MM pH 6.0 for preculture and subculture. The supernatant of 72 h subculture was analyzed on the CCM and PCA production.

### 4.9 Nanopore sequencing

Cells of CCM-Con, CCM-Min, CCM-Max, PCA-Con, PCA-Min, and PCA-Max groups (3 replicates each) for plasmid extraction were inoculated from glycerol stock and cultured overnight in 2.5 mL MM in 14 mL culture tube. Plasmid extraction was performed with Zymoprep Yeast Plasmid Miniprep II (Zymo research). The resulting plasmid samples were individually used as the templates (1 μL for 50 μL reaction mix) for a PCR with primer sets of 23376/23377 and 24990/23436 for CCM and PCA samples respectively, using Phusion High-Fidelity PCR Master Mix with HF Buffer (Thermo Scientific). After a 20-cycle amplification, the PCR products (343 bp and 425 bp for CCM and PCA samples respectively) were purified using AMPure XP beads (Beckman Coulter) and were then subjected to the sample preparation using the PCR Barcoding Kit (SQK-PBK004, (Oxford Nanopore Technologies)) for the sequencing.

The sample preparation was performed following the manufacturer's instruction. Briefly, for each sample, 100 ng PCR product was end-repaired, dA-tail-added, and ligated with the PCR adaptors (Barcode Adaptors). The resulting sample was subjected to a 14-cycle PCR with Barcode Primers, with different barcodes used for each separate sample. These barcoded libraries were purified separately and pooled in the equal molar ratio: 9 samples for the CCM group and eight samples for the PCA group, respectively. For each of the CCM and PCA groups, 200 fmol DNA of the pooled libraries were ligated with the Rapid adaptor and sequenced with a SpotON Flow Cell (R9.4 (Oxford Nanopore Technologies)). Raw sequence data in fast5 format was collected for 8 h.

### 4.10 Sequence data processing

To relate the read number to specific transporter genes (count generation), we first converted the raw reads recording the electronic signal for the sequencing in fast5 format to reads for DNA sequence in fastq format with Albacore v2.3.3 (Oxford Nanopore Technologies). The reads from pooled samples were then sorted according to the different barcode sequences. Only basecalled reads with the correct barcode were used for the subsequent mapping to the *Saccharomyces cerevisiae* genome using BWA v0.7.17 (Li and Durbin, 2010). The mapped reads were sorted with Samtools v1.9 (Li et al., 2009b) and the read for specific genes were counted with bedtools v2.27.1 (Quinlan and Hall, 2010).

The frequency of each gene was calculated by dividing the read count by the total experimental counts for each sample (Roy et al., 2018). The frequency change for each gene was calculated as enrichment score: frequency in the sorted sample/frequency in the corresponding control sample. Statistical difference between control and test groups was determined by two-tailed Student's *t*-test. In addition, the enriched genes were also analyzed following an algorithm for differentially expressed genes using DEseq2 (Love et al., 2014).

### 4.11 Transporter assay with oocyte cells

The *Xenopus laevis* oocytes (Ecocyte Bioscience) were maintained at 18 °C in Kulori buffer (90 mM NaCl, 1 mM KCl, 1 mM MgCl_2_, 1 mM CaCl_2_, 10 mM HEPES) pH7.4. Transporter genes were first cloned into the plasmid pCFB5245, and the resulting plasmid was used as the PCR template for the expression cassette fragment (T7p-β-globin 5-UTR-transporter ORF-β-globin 3-UTR). The fragment was then used as the template to synthesize the capped RNAs for transporter expression in oocytes using the T7 mMESSAGE mMACHINE™ kit (Ambion). The synthesized cRNAs (> 500 ng/μL) with high quality- very few or no noise peak determined using Agilent 2100 Bioanalyzer (Agilent Technologies), were further used.

50 nL of the cRNAs for transporter expression (400 ng/μL transporter cRNA and 100 ng/μL GFP cRNA) and control (100 ng/μL GFP cRNA) was injected into oocytes using an automatic injection system (RoboInject, Multi Channel Systems) 3 days prior to transport assays (Darbani et al., 2019). After 3 days incubation at 18 °C, cells were measured on the GFP fluorescence, and only the ones with fluorescence (compared to the blank cells) were grouped for biological replicates (10 cells each) in the further test.

For the uptake test, cells were pre-incubated in Kulori buffer pH5.6 for 5 min and then transferred into Kulori buffer pH5.6 with 100 mg/L PCA and 100 mg/L CCM for incubation at 18 °C for 130 min. For the injection test, cells were injected with 50 nL compound solution (100 mg/L PCA and 100 mg/L CCM, pH 5.5), washed with Kulori buffer pH7.4 for three times and then incubated at 18 °C for 3 h. After the incubation for both uptake test and export assay test, oocytes were washed with Kulori buffer pH7.4 for three times and then broken in 100 μL 50 % methanol and stored at 4 °C overnight. 70 μL supernatant of the cell extract was mixed with 50 μL H2O and then subjected to the LC-MS analysis.

LC-MS was performed on EVOQ EliteTriple Quadrupole Mass Spectrometer system coupled with an Advance UHPLC pump (Bruker, Fremont Ca), with the samples being held at 5 °C during the analysis. 1 μL sample was injected onto an ACQUITY HSS T3 (100 mm × 2.1 mm, 1.8 μm) C18 UHPLC column (Waters), held at a 30 °C.

The solvent system used was solvent A “MilliQ water with 0.1 % formic acid” and solvent B “acetonitrile with 0.1 % formic acid”. The flow rate was 0.4 mL/min with an initial solvent composition of %A = 100, %B = 0 held until 0.50 min, the solvent composition was then changed following a linear gradient until it reached %A = 5.0 and %B = 95.0 at 1.00 min. This was held until 1.79 min when the solvent was returned to the initial conditions and the column was re-equilibrated until 4.00 min.

The column eluent flowed directly into the Heated ESI probe of the MS, which was held at 250 °C and a voltage of 2500 V. MRM Data was collected in negative ion mode. The target masses for CCM and PCA are shown in Table S10. The other MS settings were as follows, Sheath Gas Flow Rate of 50 units, Nebuliser Gas Flow Rate of 50 units, Cone Gas Flow Rate of 20 units Cone Temp was 350 °C, and collision gas pressure 1 mTorr.

### 4.12 Yeast cell screens for betaxanthins transporters

Transporter disruption yeast libraries for betaxanthins transporters were generated by transforming Sctrans-PP into ST10362 (CEN.PK background strain) or ST10366 (S288c background strain). The resulting two yeast libraries were plated onto MM or MM + Leu + His + Met, with ca. 1000 colonies on each square petri dish. After three-day and four-day growth at 30 °C, variant strains with higher yellow color intensity in the colony were selected for strain evaluation. ST10362- and ST10366-derived variant strains were inoculated into 500 μL SD and SD-Ura media in 96-deep well plate, respectively. The cells were cultivated for 20 h for pre-cultivation. The resulting preculture was then sub-inoculated into 600 μL SD and SD-Ura media, with the initial OD_600_ as 0.05, and sub-cultivated for 24 h before the OD_600_ and betaxanthins fluorescence quantification.

Extracellular fluorescence was measured on the supernatant after centrifuging the cell culture for 5 min at 4000 rpm. The cell pellet was washed with 1 mL water and resuspended in 600 μL water. The resulting cell sample was diluted 10-time for the OD_600_ and intracellular betaxanthins fluorescence quantification.

The strains pre-selected based on the 1^st^ round evaluation were cultivated and re-validated following the same procedures described above, with three biological replicates.

## Data availability

The raw Miseq data and raw Nanopore sequencing data have been deposited in the European Nucleotide Archive, under the individual accession no. PRJEB39414 and PRJEB34026, respectively.

## Author contributions

IB and GW conceived the study and designed the experiments. GW, IMH, MB, VD, HBC, and BD performed the experiments. GW and IB analyzed the data and wrote the manuscript. MKJ and IB supervised the project. All authors have read, edited, and approved the final manuscript.

## Declaration of competing interest

The authors declare no competing interests.
