# Supplementary material for: Transportome-wide engineering of Saccharomyces cerevisiae
Source: Metab Eng. 2021 Mar;64:52–63. doi: 10.1016/j.ymben.2021.01.007 (PMC7970624; doi:10.1016/j.ymben.2021.01.007)
Supplement: Multimedia component 1 [file mmc1.docx]

Supplementary Information for

**Transportome-wide engineering of *Saccharomyces cerevisiae***

Guokun Wang^1^, Iben Møller-Hansen^1^, Mahsa Babaei^1^, Vasil D'ambrosio^1^, Hanne Bjerre Christensen^1^, Behrooz Darbani^1^, Michael Krogh Jensen^1^, Irina Borodina^1, *^

^1^ The Novo Nordisk Foundation Center for Biosustainability, Technical University of Denmark, 2800 Kongens Lyngby, Denmark

*Correspondence:

Dr. Irina Borodina,

The Novo Nordisk Foundation Center for Biosustainability, Technical University of Denmark,

Kemitorvet Building 220, 2800 Kongens Lyngby, Denmark.

irbo@biosustain.dtu.dk


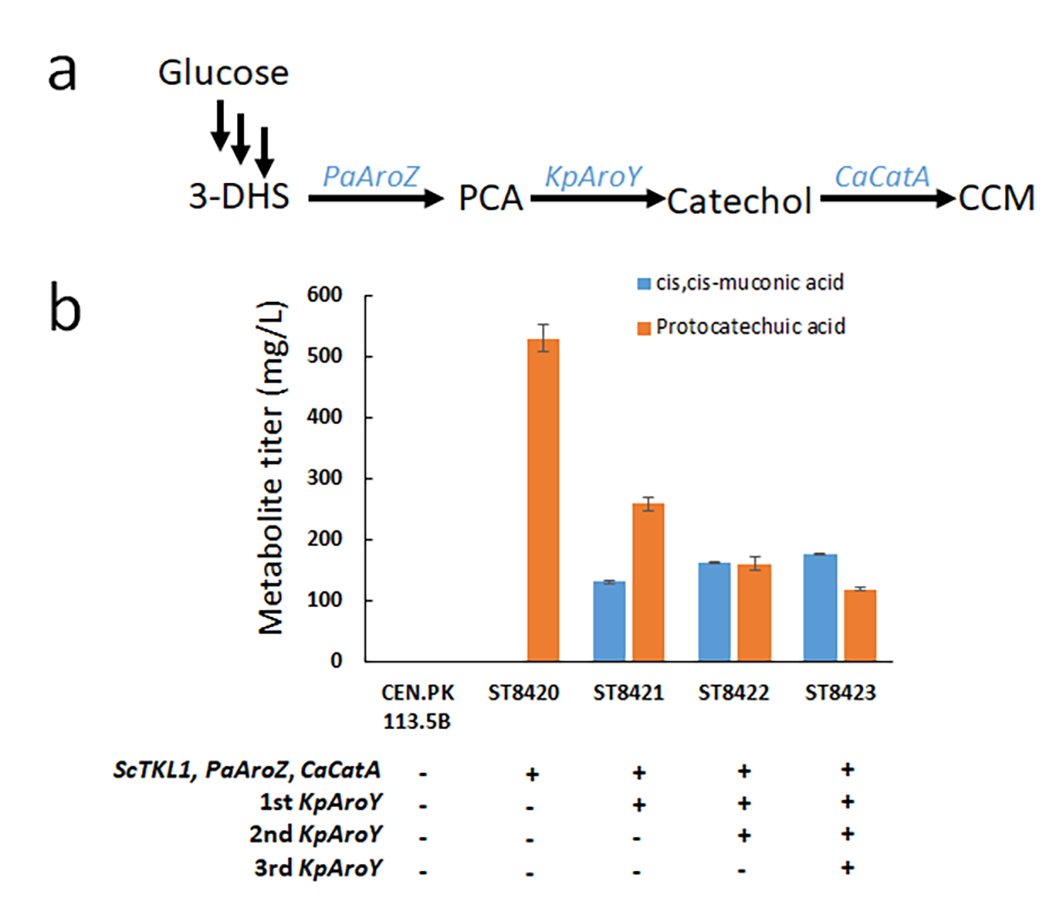


Figure S1. The production profiles of muconic acid and protocatechuic acid of engineered strains

(a) Metabolic pathway for muconic acid biosynthesis in *S. cerevisiae*, blue characters indicate the heterologous genes and multiple arrows indicate multiple reactions; (b) the various production level of muconic acid and protocatechuic acid in the strains rationally engineered. Data shown are mean values ± SDs of biological triplicates.

3-DHS: 3-dehydroshikimate, PCA: protocatechuic acid, CCM: *cis*,*cis*-muconic acid


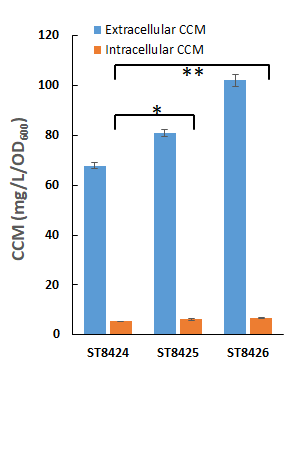


Figure S2 CCM production of engineered strains

Strains were cultivated on mineral medium pH 6.0 supplemented with 20 mg/L uracil and test performed after 72 h. Data shown are mean values ± SDs of biological triplicates. Statistical difference was determined by two-tailed Student’s *t* test. **P* < 0.05; ***P* < 0.01.


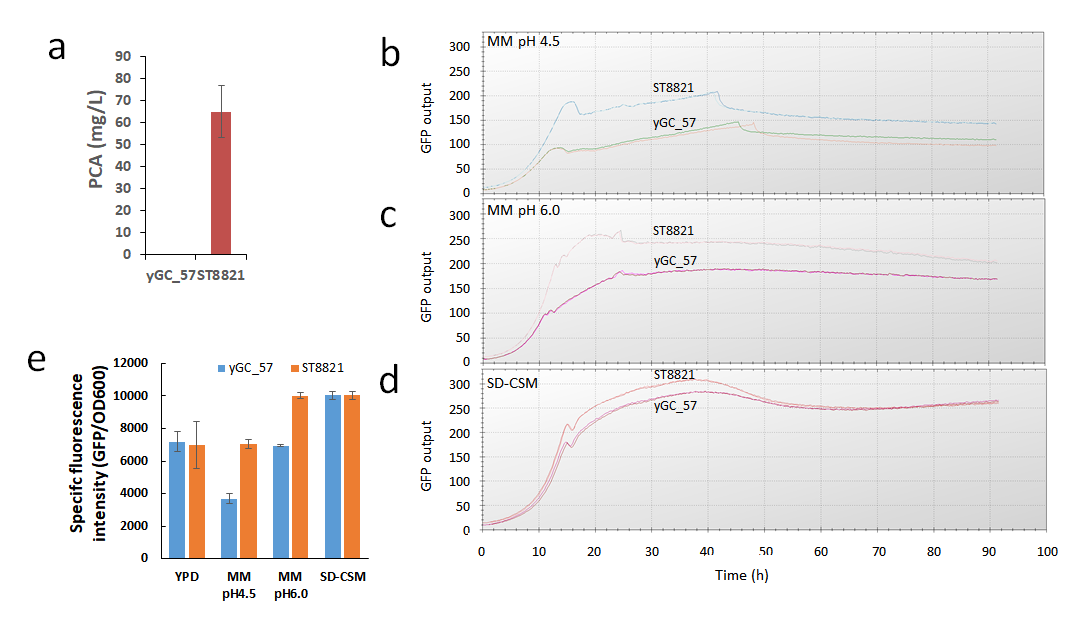


Figure S3 Fluorescence output of PCA-responsive PcaQ biosensor in a PCA producing strain

PcaQ biosensor-contained strains, yGC_57 (control strain) and ST8821 (PCA producing strain) were cultured on YPD for quantification of PCA titers (a), and were cultivated on different media to validate the optimal cultivation condition and timepoint for detecting the time-course PCA responsive fluorescence. Mineral media (MM) at pH 4.5 (b) or pH 6.0 (c) supplemented with 20 mg/L uracil and SD-CSM medium (d) were used for the test. The PCA-responsive fluorescence output was verified further on 24 h culture with microplate reader (e).

Only on MM, the two strains showed clearly different fluorescence output, indicating MM as the sound condition for resolving the PCA responsive fluorescence increase. The background fluorescence on MM with pH 4.5 was low, and, therefore, it was selected as the condition for further study.

In this work, the PCA biosensor underperformed the CCM biosensor in giving compound-responsive fluorescence. The possible reason is that the CCM biosensor used in this work has been well optimized regarding the activator protein (BenM) sequence, effector promoters, and the position of the binding site of BenM in the promoter. In contrast to this, the development of the PCA biosensor is at an early stage. In this work, we employed a design that needs stringent cultivation conditions to trigger the fluorescence change.

Data shown are GFP output value of two samples for each test (b, c and d) or mean values ± SDs of biological triplicates (a and e).


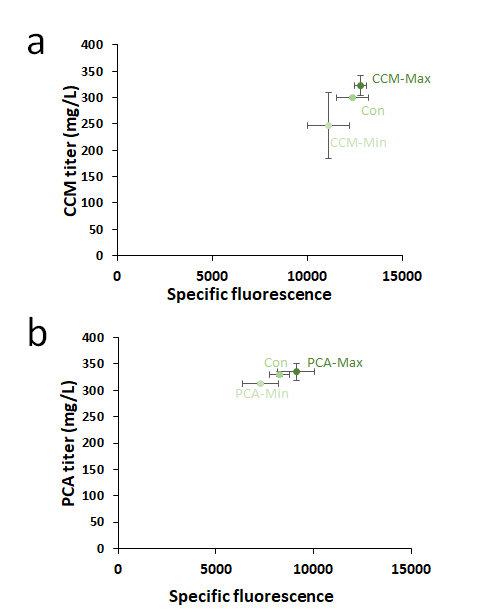


Figure S4 The production of CCM or PCA of sorted cell pool from the transporter disrupted yeast cell libraries.

The cell pool with the highest fluorescence (Max) or the lowest fluorescence (Min) of the yeast cell libraries constructed based on ST8424 (for CCM) or ST8859 (for PCA) were evaluated. ST8424 and ST8859 with empty plasmid were used as the controls, CCM-Con and PCA-Con, respectively. Specific fluorescence and metabolite were detected on 24 h and 72 h culture respectively. Data shown are mean values ± SDs of biological triplicates.


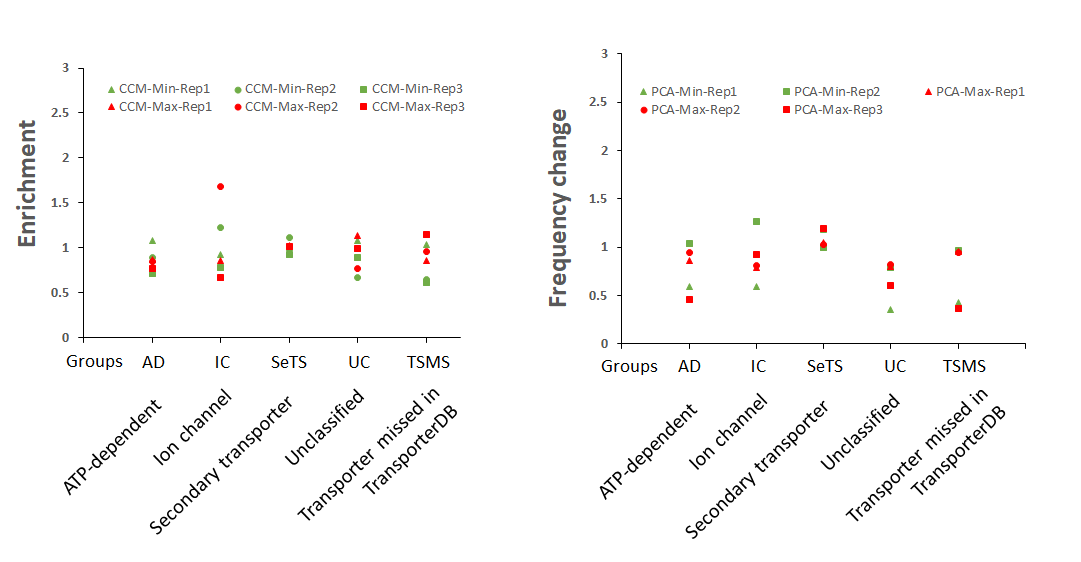


Figure S5 Frequency change for transporters in sub-groups

Frequency changes of transporters in each sub-groups after cell sorting were shown as the fold change of read ratio to the control samples. Each dot corresponds to a frequency change value of a group of transporters in an individual sample/replicate.


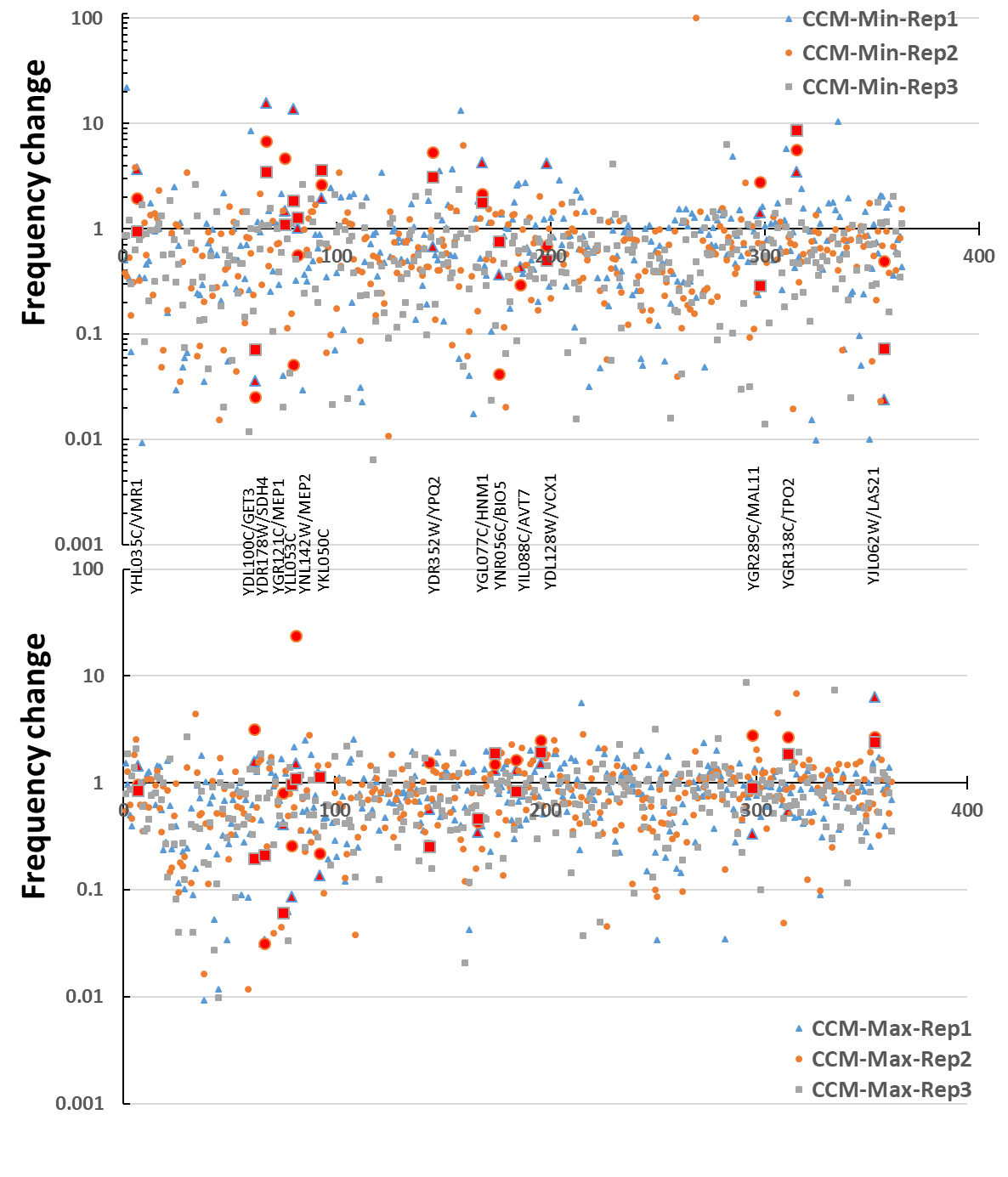


Figure S6 Changes in the frequencies of enriched transporters for CCM by cell sorting

The frequency of each transporter was defined as the ratio of the read count to the total experimental counts for each sample. Frequency change for each genes was compared with the control samples. Data shown from biological triplicates individually for each transporters.

The genes selected for experimental validation were marked as symbols (•, ▲, and ▇) in a bigger size in red.


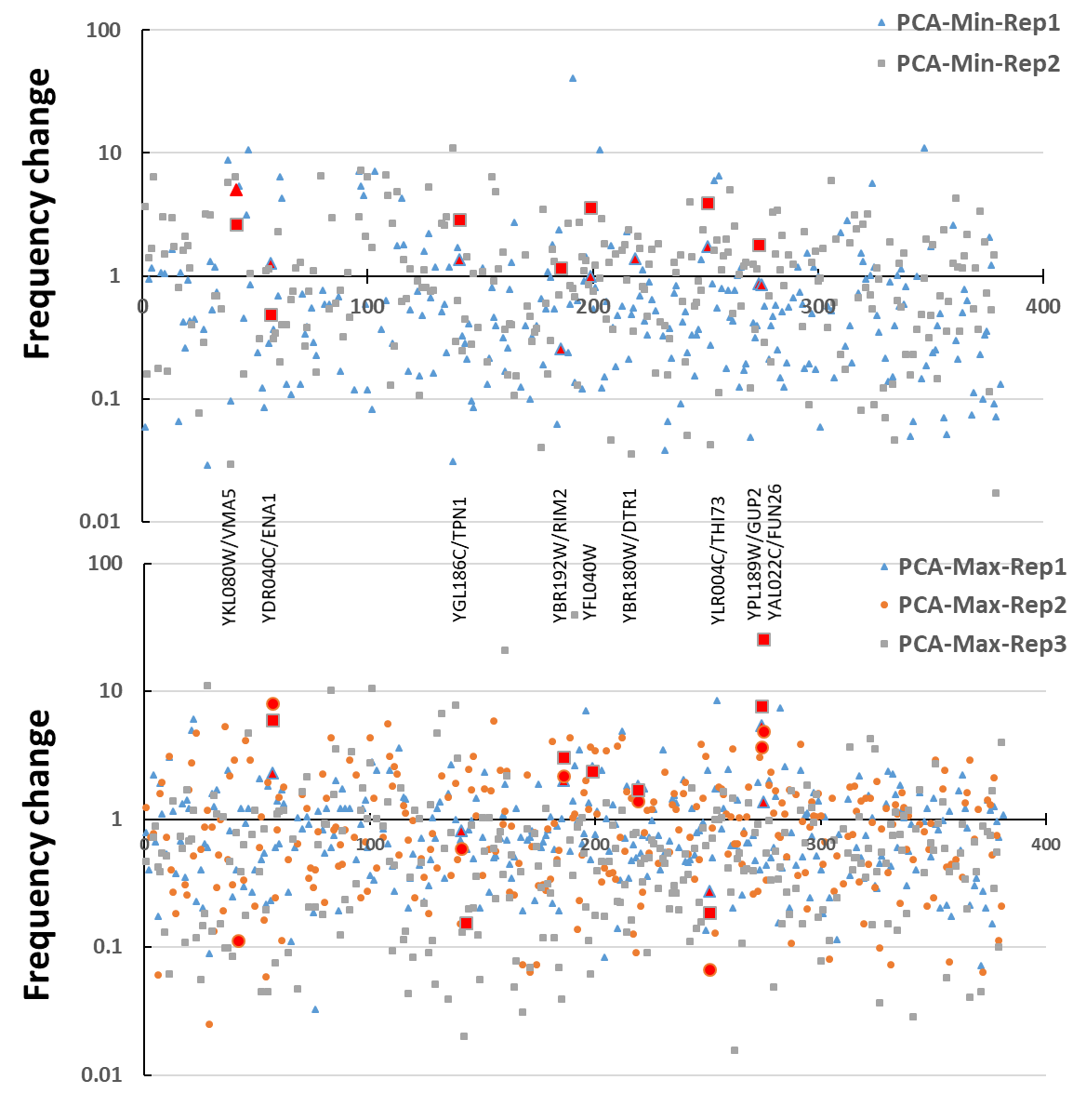


Figure S7 Changes in the frequencies of enriched transporters for PCA by cell sorting

The frequency of each transporter was defined as the ratio of the read count to the total experimental counts for each sample. Frequency change for each genes was compared with the control samples. Data shown from biological duplicates (PCA-Min) or triplicates (PCA-Max) individually for each transporters.

The genes selected for experimental validation were marked as symbols (•, ▲, and ▇) in a bigger size in red.


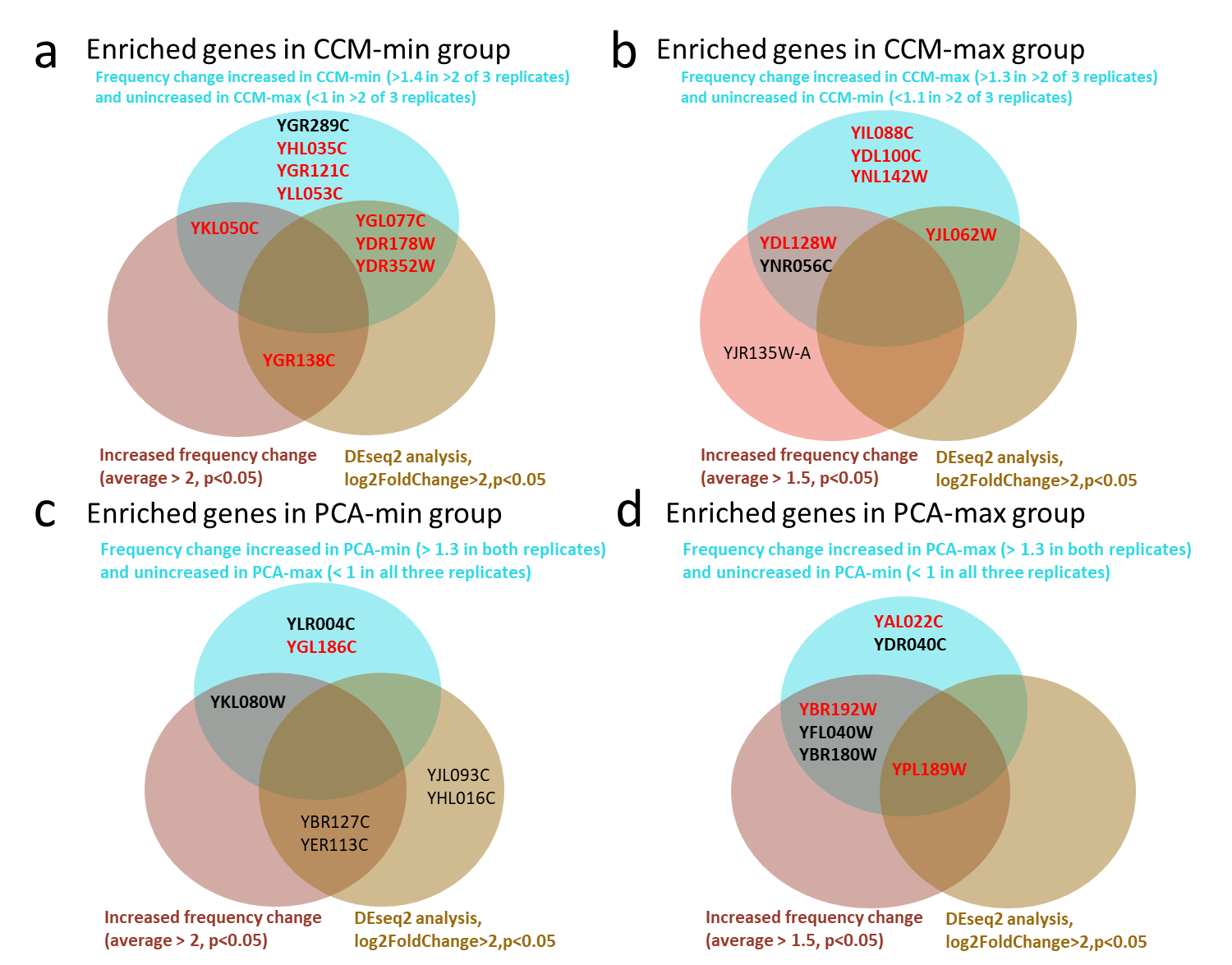


Figure S8 Gene selection for reverse validation

Enriched genes in each screen were analyzed on the frequency change (turquoise or dark red circles) or DEseq2 (dark yellow circle). Genes in bold font were selected for reverse validation, and those in red color were the functional ones.


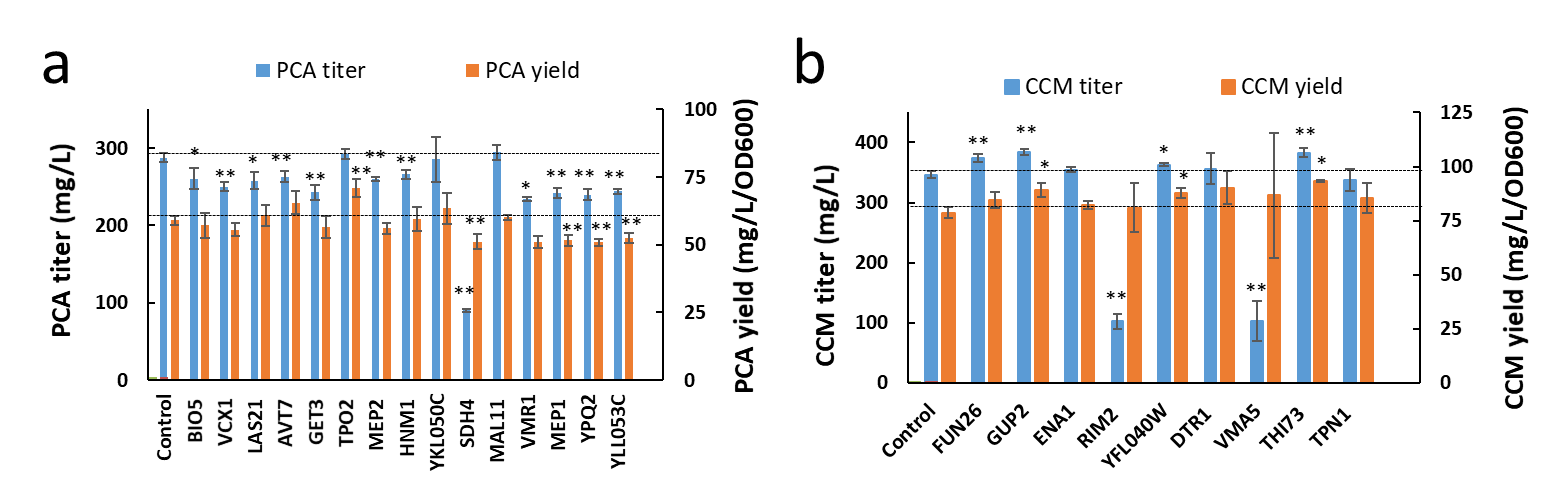


Figure S9 The PCA (a) or CCM (b) production pattern of strains reverse engineered with the transporter disruption for CCM (a) or PCA (b). ST8424 and ST8859 were used as the parental strain respectively and metabolite was quantified on 72 h culture.

Data shown are mean values ± SDs of biological triplicates. Statistical difference between control and indicated strains was determined by two-tailed Student’s *t* test. **P* < 0.05; ***P* < 0.01.


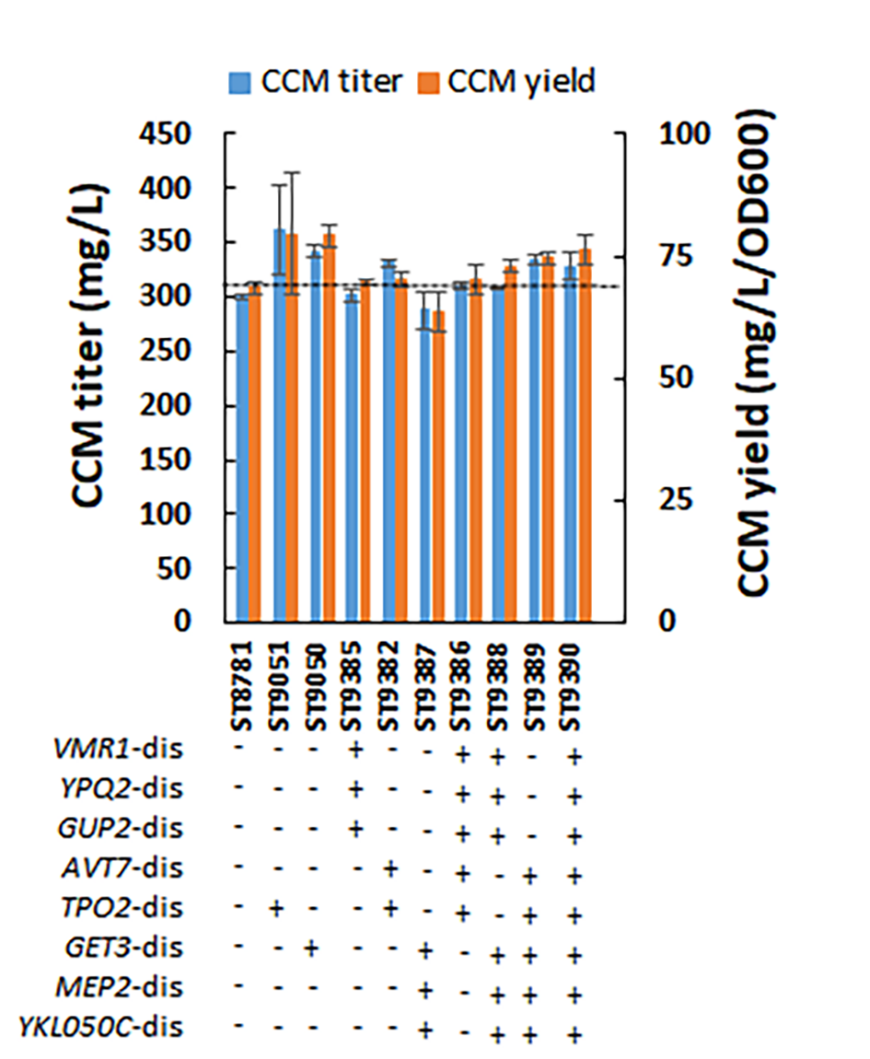


Figure S10 The CCM production by strains with multiple transporter disruptions. ST8424 was used as the parental strain and metabolite was quantified on 72 h culture.

Data shown are mean values ± SDs of biological triplicates.


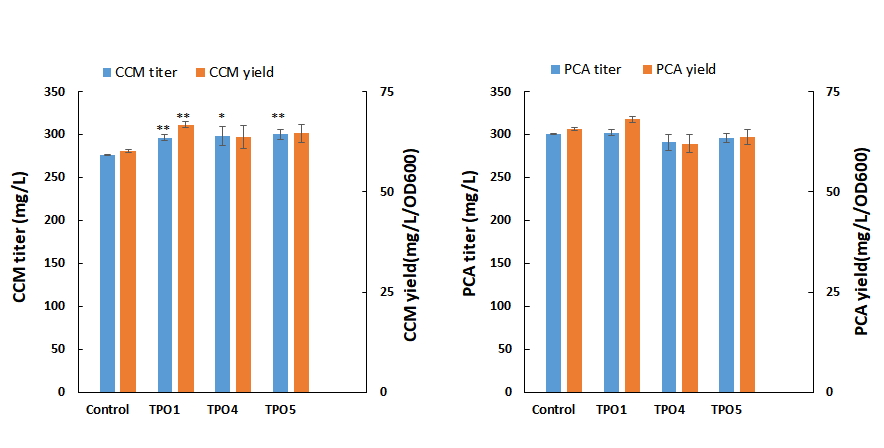


Figure S11 The CCM and PCA production by strains with transporter of polyamine disrupted. ST8424 was used as the parental strain and metabolite was quantified on 72 h culture.

Data shown are mean values ± SDs of biological triplicates. Statistical difference between control and indicated strains was determined by two-tailed Student’s *t* test. **P* < 0.05; ***P* < 0.01.


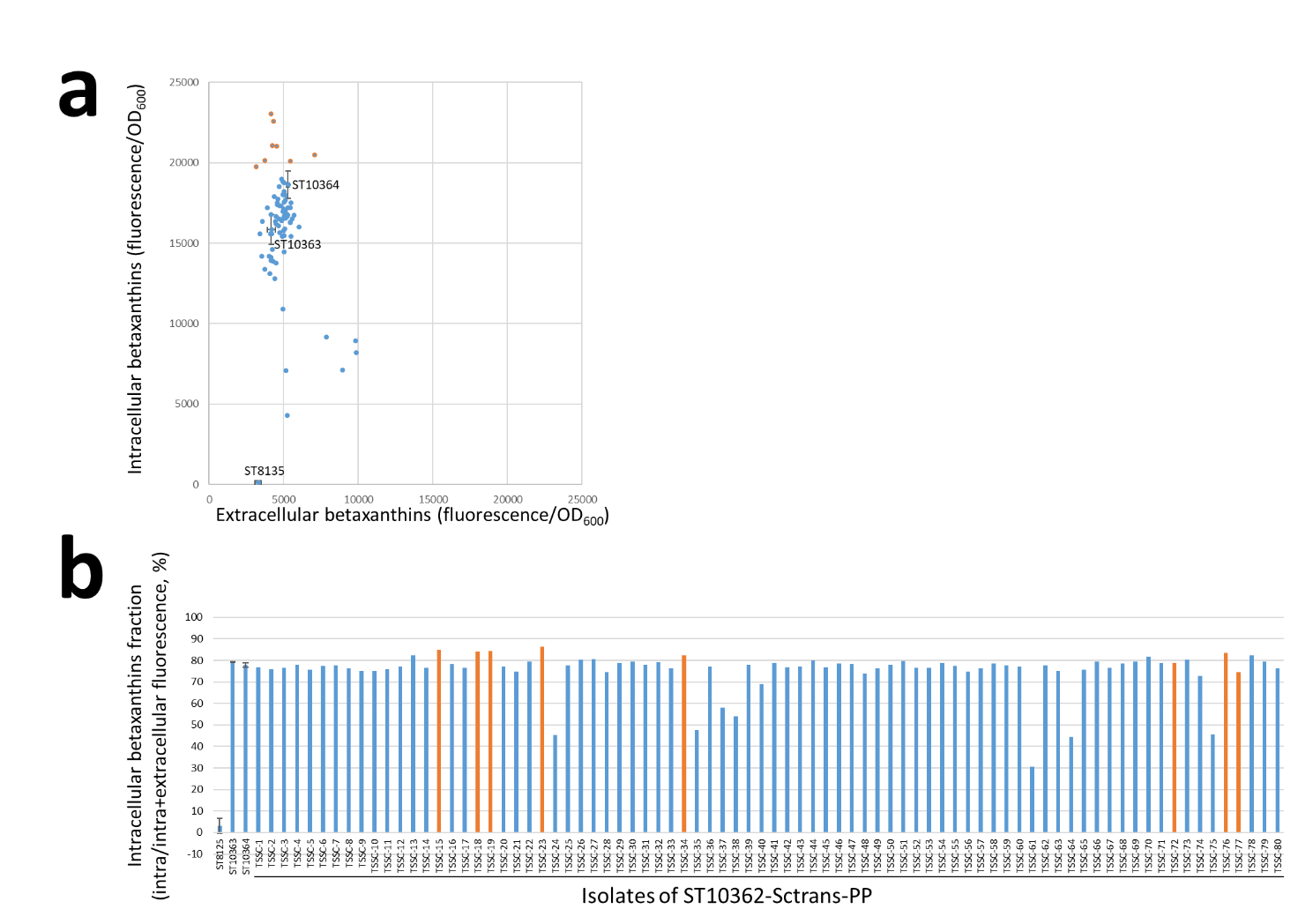


Figure S12 The betaxanthins production (a) and distribution (b) pattern of variant strains with the transporter disruption.

ST8135 is a CEN.PK background strain carrying genome integrated Cas9. ST10362 is a ST8135 derived strain that harbors betaxanthins biosynthesis pathway and *URA3* knockout. ST10363 and ST10364 are ST10362 carrying empty plasmid and *QDR2* disruption, respectively.

*QDR2* encodes a quinidine/cations-related transporter, and its deletion increased the intracellular betaxanthins fraction in S288c background strain. Here *QDR2* disruption was designed as the positive control which is expected to show improved intracellular betaxanthins. Surprisingly *QDR2* disruption didn’t impact the betaxanthins distribution in this test, likely due to the different strain background.

Variant strains derived from ST10362 were selected for higher color intensity in colonies and subjected to the cultivation for strain evaluation. Betaxanthins were quantified on 24 h sub-culture in synthetic medium without amino acid and ammonia sulfate.

Data shown for ST8135, ST10363, and ST10364, are mean values ± SDs of biological triplicates. Data shown for variant strains are from single replicates.

Orange dots (a) and bars (b) indicated the eight strains with the highest intracellular betaxanthins fluorescence. These strains were selected for further validation and transporter identification.


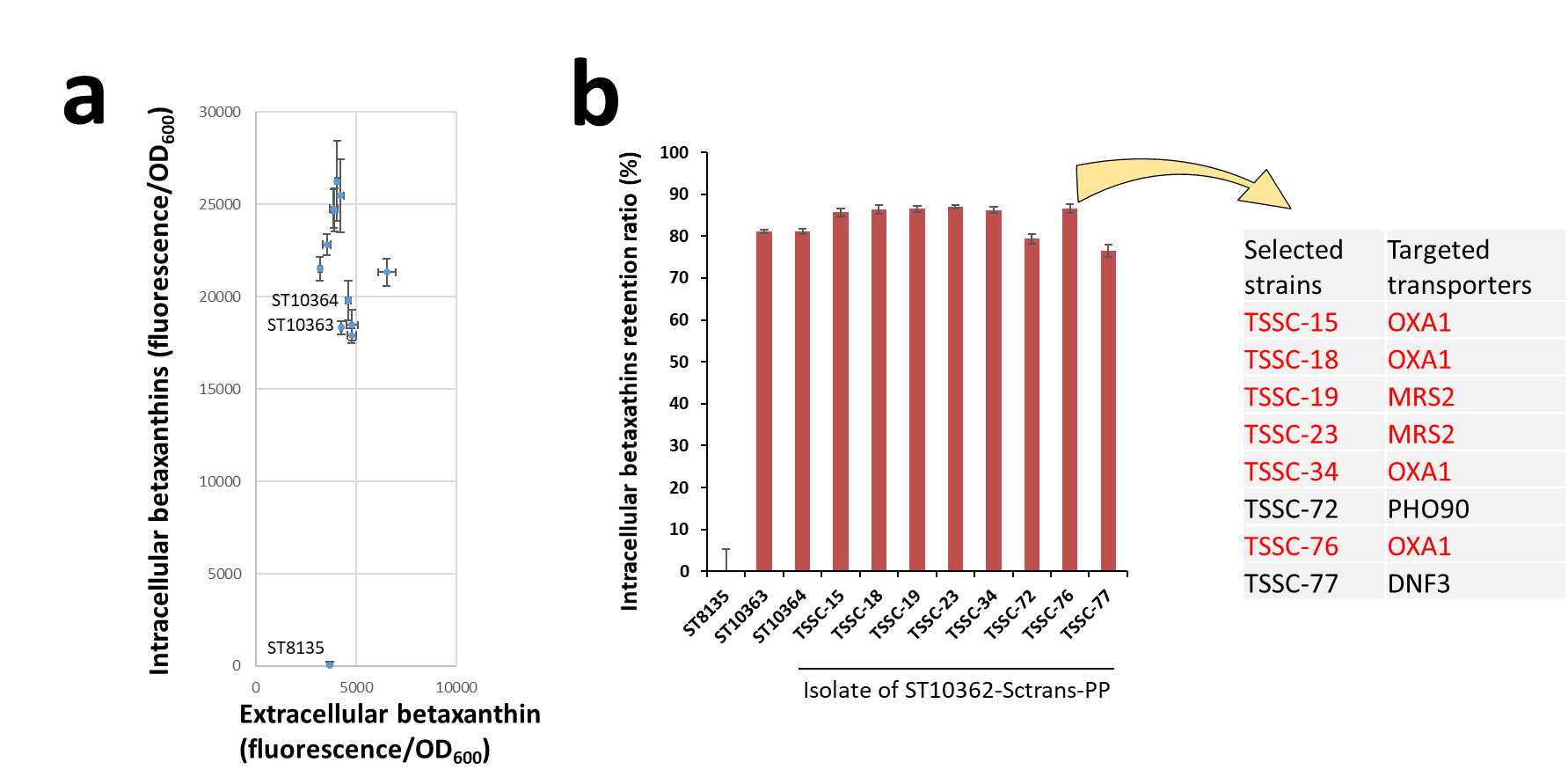


Figure S13 The betaxanthins production (a) and distribution (b) pattern of pre-selected variant strains with the transporter disruption.

Variant strains of CEN. PK background strains, pre-selected for higher cellular betaxanthins retention, were subjected to the strain validation in three biological replicates and transporter identification. ST8135 is a CEN.PK background strain carrying genome integrated Cas9. ST10362 is a ST8135 derived strain that harbors betaxanthins biosynthesis pathway and *URA3* knockout. ST10363 and ST10364 are ST10362 carrying empty plasmid and *QDR2* disruption, respectively. ST8135 serves as a non-betaxanthins-producing control strain, and ST10363 acts as the betaxanthins-producing control strain.

*QDR2* disruption was designed as the positive control which is expected to show improved intracellular betaxanthins. However, *QDR2* disruption didn’t impact the betaxanthins distribution in this test, likely due to the different strain background (CEN.PK background strain vs. S288c background strain that is used in the literature).

Betaxanthins were quantified on 24 h sub-culture in synthetic medium without amino acid and ammonia sulfate. Data shown are mean values ± SDs of biological triplicates, and data in both panels a and b are from one experiment.

Disrupted transporters were verified through the Sanger sequencing of the PCR product for sgRNA-donor region in the plasmid. Red font in panel b indicates transporters identified in variant strains that showed higher intracellular betaxanthins retention ratio in the validation.


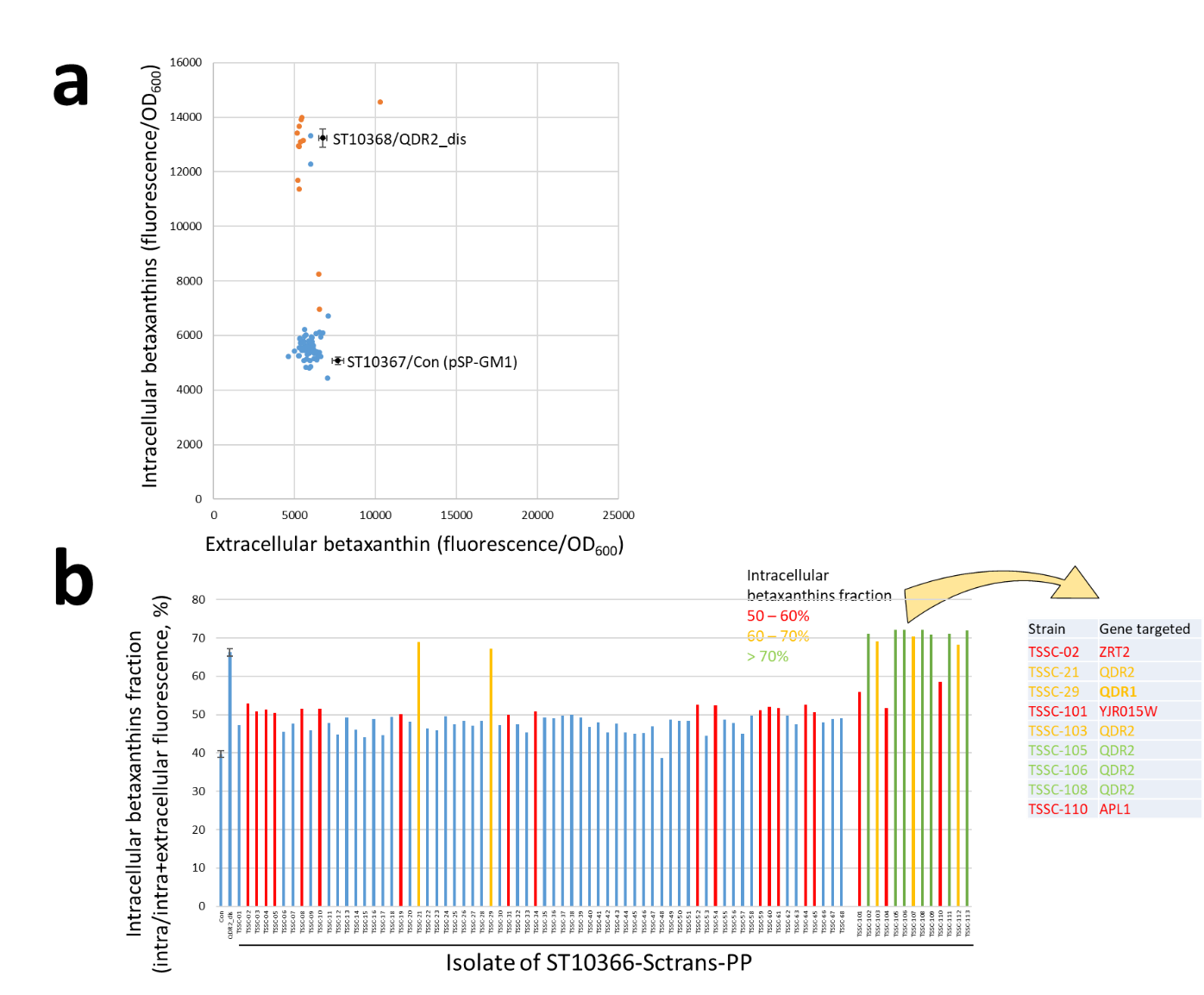


Figure S14 The betaxanthins production (a) and distribution (b) pattern of variant strains with the transporter disruption.

ST10366 is a BY4741 background strain carrying genome integrated Cas9, betaxanthins biosynthesis pathway, and *URA3* knockout. ST10367 and ST10368 are ST10366 carrying empty plasmid and *QDR2* disruption, respectively. *QDR2* disruption was designed as the positive control which is expected to show an improved intracellular betaxanthins.

Variant strains derived from ST10366 were selected for higher color intensity in colonies after transformation for 3-day and 4-day, with blue and orange dots (a) indicated, respectively. Those variant strain isolates were subjected to the cultivation for strain evaluation. Betaxanthins were quantified on 24 h sub-culture in synthetic medium without uracil. Data shown for ST10367 and ST10368 are mean values ± SDs of biological triplicates, whereas data for variant strains are from single replicates.

The variant strain isolates that showed higher cellular betaxanthins fraction were selected for transporter identification, with three random strains for each range: 50 – 60%, 60 – 70%, and > 70%. Disrupted transporters were verified through the Sanger sequencing of the PCR product for sgRNA-donor region in the plasmid.
